# A simulation-based approach to strengthen chronic wasting disease surveillance in captive cervid populations

**DOI:** 10.1371/journal.pone.0350825

**Published:** 2026-06-24

**Authors:** Lauren Wakefield, Alan Cain, Chris Cerny, Hunter Reed, Aniruddha V. Belsare

**Affiliations:** 1 Auburn University College of Forestry, Wildlife and Environment, Auburn, Alabama, United States of America; 2 Texas Parks and Wildlife Department, Austin, Texas, United States of America; 3 Auburn University College of Veterinary Medicine, Auburn, Alabama, United States of America; Incheon National University, KOREA, REPUBLIC OF

## Abstract

Captive cervid facilities are at elevated risk for chronic wasting disease (CWD) due to high deer densities, close animal contact, environmental contamination, and frequent movement of deer between facilities and across regions. Once CWD becomes established, it spreads quickly and is nearly impossible to eliminate, making early detection critical. However, surveillance in captive herds is challenging: testing only a small portion of the herd during the early stages of CWD outbreak provides a low likelihood of detecting infected deer. Moreover, not detecting CWD in a small sample cannot be interpreted as proof that the facility is free of CWD unless the entire herd is tested using a diagnostic test with 100% sensitivity. We developed a simulation-based approach to interpret surveillance results when CWD is not detected in the sampled subset of animals within captive cervid facilities. This simulation-based approach is retrospective in nature. By integrating deer demographic data, individual movement histories, and past CWD testing records, this tool estimates facility-level CWD detection probabilities, providing a clearer assessment of the likelihood of undetected disease within a facility. Using data from 23 captive cervid facilities in Texas, we demonstrate how this tool can support risk-based monitoring and help wildlife agencies prioritize surveillance and management efforts where they are most needed.

## Introduction

Chronic wasting disease (CWD) is a progressive, invariably fatal, neurodegenerative disease of cervids caused by misfolded forms of the normal host cellular prion protein. A highly contagious transmissible spongiform encephalopathy (TSE), CWD is transmitted through both direct contact between animals as well as indirect contact via contaminated environment and fomites. CWD circulates in both wild and captive cervid populations across North America [1]. CWD has also been detected in free-ranging reindeer (*Rangifer tarandus*) in Norway and in captive cervids in South Korea. Moreover, a novel form of CWD with atypical characteristics has been reported in moose and red deer in Norway, as well as in moose in Finland and Sweden [2–5]. These cases appear to represent sporadic CWD (sCWD), with little or no evidence of transmission between live animals. Several risk factors contribute to CWD risk in captive cervid facilities, including artificially high deer densities, close quarters and regular contact between animals for long periods of time, exposure to the contaminated environment, and the movement of captive cervids across geographic regions [6–8]. Among these, the spread of CWD via human-facilitated movement of live captive cervids is one of the mechanisms responsible for the spread of CWD into new regions, including previously unaffected states and countries [6,9]. For example, CWD-infected elk (*Cervus canadensis*) from the US state of South Dakota are believed to have initiated the outbreak in 39 farmed elk herds in the Canadian province of Saskatchewan between 1996 and 2002 [10]. Similarly, the export of CWD-infected elk from Canada to South Korea in 2001 led to the establishment of CWD in captive elk populations there [11]. Moreover, there is a persistent risk of reciprocal CWD transmission between captive and wild cervids, because fences are permeable [7].

Introduction of CWD into a captive cervid facility may lead to rapid disease spread, resulting in high infection rates and in some cases, prevalence nearing 100% [8,12–14]. Compounding this challenge is the disease’s long pre-clinical phase, during which the infected animal exhibits no outward signs of disease but continues to shed prions into the environment, resulting in the contamination of shared resources such as feed, water, soil, and surfaces, creating reservoirs of infectivity within the facility. As a result, even after removal of infected individuals, prions can remain viable in the environment for years, posing a continuous risk to susceptible animals. The combination of prolonged silent transmission, environmental persistence, and the absence of preventive (vaccine) or curative measures underscores the critical importance of robust, proactive surveillance systems that can detect CWD in its earliest stages, enabling timely intervention to prevent the establishment of CWD in captive and wild cervid populations [15,16].

The United States Department of Agriculture (USDA) implemented a CWD Herd Certification Program (HCP) in 2014 to ensure comprehensive surveillance of captive herds and premises, aiming to prevent the commercial movement of infected farmed cervids. The CWD Herd Certification Program is voluntary; however, herd certification is required for the interstate movement of captive cervids [17]. A key requirement of this program is that all on-farm deaths of cervids aged 12 months or older must be tested for CWD. Five consecutive years of CWD-free testing is required to certify herds as being low risk for having CWD, and animals from such herds may be shipped interstate. However, this requirement can sometimes result in a small number of animals being tested, even no animals tested at all, particularly in herds with small population sizes, low mortality rates, or frequent movement of animals in and out of the herd without an opportunity for testing. CWD has been detected in HCP certified herds, and transmission through intra- and interstate movement of animals from HCP certified herds to other facilities has been documented [18]. Individual states may implement regulations that are more stringent than the HCP requirements. For example, in Texas, in addition to requiring CWD testing of all mortalities within captive cervid facilities, ante-mortem testing is also mandated for any deer being moved from a facility.

CWD diagnosis primarily relies on postmortem detection of abnormal prion proteins in retropharyngeal lymph nodes and brainstem tissue using immunohistochemistry (IHC), enzyme-linked immunosorbent assay (ELISA), or western blotting, although approved routine diagnostic testing is currently limited to IHC and ELISA [17]. Ante-mortem testing of peripheral lymphoid tissues, particularly tonsil and recto-anal mucosa-associated lymphoid tissue (RAMALT) by immunohistochemistry can improve the probability of detecting CWD infected animals before death, especially during the early stages of infection [19–21]. More recently, amplification assays such as protein misfolding cyclic amplification (PMCA) and real-time quaking-induced conversion (RT-QuIC) have demonstrated substantially greater sensitivity than conventional diagnostic methods, allowing detection of very low concentrations of prions in tissues and body fluids [22]. These approaches may permit earlier detection of CWD during the preclinical phase of infection and from alternative sample types collected before or after death, thereby improving the likelihood of detecting infected animals when prevalence is low or infection is recent. At present, no diagnostic test can reliably determine that an individual animal is free of CWD. Existing tests are not sufficiently sensitive to detect all infected animals, particularly during the early stages of infection, because CWD has a prolonged incubation period that can result in false-negative test outcomes before prion accumulation reaches detectable levels.

Regardless of the testing method, an important issue is the sample size, or the proportion of individuals tested from the population. This is particularly critical during the pre-establishment phase of CWD outbreak, when only a few animals may be infected and are still in the early pre-clinical phase. Sample sizes are often insufficient to confidently detect CWD in such low-prevalence scenarios [15,16]. Testing a small subset of the herd during the pre-establishment phase of CWD outbreak yields a low detection probability, as the likelihood of including an infected deer in the sample is minimal. Therefore, CWD not detected test results from a herd cannot be interpreted as confirmation of disease absence, unless the entire herd is tested with a 100% sensitive test. Such a high intensity testing scenario with a high sensitivity diagnostic test is rarely feasible or sustainable. What is needed, then, is a practicable strategy for interpreting CWD not detected test results from a subset of the herd – one that accounts for herd size, individual deer domiciliary history, and CWD testing records. Such an approach can serve as a practical tool for assessing and communicating the risk of undetected CWD in captive cervid facilities.

Here, we present a simulation-based approach for estimating facility-level CWD detection probability. Individual deer histories and multiyear CWD testing data are incorporated into the process of estimating CWD detection probability. By applying the model to historical CWD testing data, facilities with a higher likelihood of undetected CWD can be identified, even when CWD is not detected in the subset of animals tested. We illustrate the application of this approach to assess the risk of undetected CWD in select captive white-tailed deer (*Odocoileus virginianus*; hereafter, deer) facilities in Texas. This approach provides regulatory agencies with a decision-support tool to standardize CWD surveillance efforts across captive cervid facilities and promote sustainable and efficient CWD surveillance strategies.

## Materials and methods

We developed an agent-based model, Cap*Ov*CWD, to simulate disease surveillance scenarios for captive cervid facilities [23]. The model was developed in NetLogo 6.4. NetLogo is a software platform for implementing agent-based models [24]. Model description is provided following the Overview, Design concepts, details (ODD) protocol for individual-based models (S1 Text). Model code is available open access via website repository Open ABM CoMSES Computational Model Library (https://doi.org/10.25937/87rx-af75). Cap*Ov*CWD was initialized using data on herd size, composition, and individual deer transfer histories obtained from captive facility records maintained by the Texas Parks and Wildlife Department (TPWD). An accounting year spans from April 1 to March 31 of the following year. Deer that were present in the facility at any point during an accounting year are included in that year’s model population, including individuals that died or were transferred in or out before the end of the year. These deer are included in the model to represent a “worst case scenario” for facilities to estimate the risk of undetected CWD in the facility. Each deer is represented as an individual agent and classified as either an adult or a fawn, with age class assigned based on the midpoint of the accounting year, when fawns from the previous year transition to the adult stage.

The number of adult and fawn deer tested for CWD in each accounting year was determined from individual testing histories, which included both ante-mortem and post-mortem test results recorded within the accounting year. In addition to the required post-mortem testing of retropharyngeal lymph nodes and brainstem tissue using IHC or ELISA, TPWD and the Texas Animal Health Commission (TAHC) have incorporated ante-mortem testing using immunohistochemistry (IHC) of samples obtained via tonsil biopsy or rectal biopsy. The diagnostic sensitivity of IHC using these tissue samples can vary widely, ranging from 25% to 95%, depending on the cervid species and genetic variability within the prion protein gene (*PRNP*) [19,21,25]. For the model implementation, diagnostic test performance was incorporated by estimating the negative predictive value (NPV) of ante-mortem testing across a range of test sensitivity values (25–70%) under a low-prevalence scenario (<1%). NPV is the measure of accuracy of a negative test result. Across this sensitivity range under a low prevalence scenario, NPV exceeded 99%, indicating a high likelihood that deer with CWD not detected ante-mortem test results were free of CWD within the limitations of currently available diagnostics. This supports the use of ante-mortem CWD not detected results as reliable indicators of CWD-negative status within the model framework. Although NPV declines at higher prevalence levels when test sensitivity is low, falling below 90% when prevalence exceeds 10%, surveillance at such prevalence levels requires substantially smaller sample sizes for confident detection of CWD, reducing the practical need for this modeling approach. The aforementioned test-derived data formed the empirical foundation for estimating facility-level CWD detection probabilities within the simulation framework. Additionally, the number of confirmed CWD-negative adults and fawns in the herd for each accounting year was calculated based on CWD not detected test results from the current or subsequent years. CWD not detected test result for a deer in a subsequent year is considered confirmed negative in prior years because CWD is a progressive and irreversible disease. Once infected, a deer cannot revert to a disease-free state or have a CWD not detected test result in later years. Therefore, within the limitations of currently available diagnostics, a CWD not detected test result indicates a high likelihood that the deer was not infected in previous years.

For each accounting year, we simulated two scenarios per age class (adults and fawns) in each captive facility. For the first scenario, sample size was defined as the number of deer in the captive facility that were tested during an accounting year and had “CWD not detected” results, based on TPWD records (Table 1). During each model iteration, one deer from the relevant age class was randomly designated as CWD-infected, and a sample of the specified size was then randomly selected for CWD testing. Annual CWD detection probability was estimated as the proportion of iterations in which the CWD-infected deer was included in the tested sample, thereby resulting in successful detection of CWD. For the second scenario, the total number of confirmed CWD-negative individuals present in the facility during the accounting year, derived from multiyear testing data, was used as the sample size to refine the annual CWD detection probability estimates (S2 Table). Why use the number of confirmed CWD-negative deer in a facility, based on lifetime testing, as the sample size to refine the detection probability estimates? A small number of CWD not detected test results only tells us that the disease was not detected in that limited subset, leaving considerable uncertainty about the rest of the herd. In contrast, the larger the group of animals confirmed negative, the lower the likelihood that CWD remains undetected in the population. By retrospectively assigning CWD negative status to animals that had CWD not detected results in later years, we can expand the pool of confirmed negatives beyond annual testing alone, providing a stronger basis for estimating detection probability and assessing disease risk in the herd. For each accounting year, 1000 iterations were conducted and summarized into 10 replicate detection probability estimates (each based on 100 iterations).

**Table 1:** Proportion of adult (A) and fawn (F) deer tested for chronic wasting disease across 23 captive cervid facilities in Texas between 2014 and 2023.

Facility ID	2014		2015		2016		2017		2018		2019		2020		2021		2022		2023	
	A	F	A	F	A	F	A	F	A	F	A	F	A	F	A	F	A	F	A	F
**TxCF1**	1/44	0/25	26/118	0/46	36/136	0/49	30/175	0/62	11/185	0/48	13/227	0/89	9/227	0/73	96/240	1/117	61/283	0/55	208/260	0/30
**TxCF2**	19/245	0/158	18/249	0/113	43/210	0/169	6/257	0/132	41/314	0/195	19/350	0/226	84/392	0/266	93/409	0/162	130/370	0/144	237/265	NF
**TxCF3**	9/166	0/72	22/231	0/136	89/320	0/150	12/374	0/150	33/494	0/106	53/511	0/171	69/547	0/390	82/574	0/357	415/684	0/383	423/477	0/129
**TxCF4**	7/123	0/16	10/68	0/60	11/87	0/25	16/72	0/36	20/85	0/32	7/61	0/14	6/53	0/27	58/72	9/27	17/84	0/22	41/61	0/19
**TxCF5**	0/259	0/137	10/209	0/132	112/335	0/193	28/463	0/239	32/476	0/315	32/406	0/256	44/477	0/332	282/511	39/263	168/559	44/210	227/288	91/103
**TxCF6**	ND	ND	ND	ND	62/127	0/79	4/43	0/36	4/97	0/72	6/115	0/91	8/167	0/78	96/191	11/99	53/166	0/63	93/166,	0/42
**TxCF7**	0/2	NF	0/2	NF	0/2	NF	0/2	NF	0/2	NF	7/86	0/53	9/131	0/44	79/199	11/141	73/322	0/51	55/216	0/41
**TxCF8**	1/82	0/14	27/133	0/6	5/100	0/33	3/124	0/20	19/170	0/42	12/225	0/66	10/141	0/45	56/167	9/46	42/138	0/21	33/59	0/21
**TxCF9**	7/243	0/169	8/218	0/184	70/210	0/163	8/199	0/148	23/172	0/129	6/166	0/168	23/168	0/97	19/147	40/90	34/159	76/163	72/221	67/125
**TxCF10**	0/108	0/40	11/130	0/35	46/132	0/9	5/86	0/21	3/86	0/35	3/100	0/32	3/112	0/36	28/110	0/36	19/129	0/29	77/140	0/30
**TxCF11**	3/155	0/75	13/205	0/99	80/211	0/97	8/267	0/150	14/299	0/155	26/345	0/103	43/308	0/103	129/264	4/115	83/262	0/115	142/292	5/126
**TxCF12**	8/181	0/58	15/171	0/83	64/194	0/71	9/193	1/89	15/190	0/80	9/180	0/95	15/219	0/62	37/205	0/80	39/182	0/61	47/189	0/27
**TxCF13**	1/337	0/94	4/372	0/105	8/356	0/121	3/380	0/159	21/440	0/186	5/462	0/196	10/575	0/249	186/710	0/224	269/790	36/253	226/850	0/240
**TxCF14**	ND	ND	0/9	0/5	1/14	0/15	1/27	0/12	2/28	0/19	1/33	0/28	2/34	0/23	11/34	0/19	8/42	0/5	8/38	0/15
**TxCF15**	17/268	0/110	14/300	0/117	50/344	0/82	29/254	0/78	19/225	0/75	16/224	0/109	28/291	0/103	131/274	0/145	131/267	3/130	32/153	0/75
**TxCF16**	0/97	0/74	4/128	0/80	53/159	0/111	28/209	0/106	7/239	0/103	14/257	0/107	27/279	0/117	30/283	0/149	67/289	0/105	23/314	0/66
**TxCF17**	16/580	0/331	12/610	0/224	110/360	0/158	26/422	0/137	18/406	0/164	9/353	0/169	15/371	0/235	355/494	0/282	199/307	58/59	ND	ND
**TxCF18**	ND	ND	ND	ND	ND	ND	ND	ND	ND	ND	ND	ND	2/148	0/159	204/355	3/89	69/277	NF	ND	ND
**TxCF19**	ND	ND	ND	ND	ND	ND	45/138	1/104	16/326	0/90	21/282	0/125	24/316	0/167	27/84	2/73	ND	ND	ND	ND
**TxCF20**	ND	ND	ND	ND	ND	ND	ND	ND	ND	ND	ND	ND	12/631	0/387	507/1126	9/554	380/1360	0/754	PF	PF
**TxCF21**	ND	ND	2/247	0/133	94/295	0/71	13/296	0/106	14/246	0/96	14/227	0/124	14/217	0/80	77/204	0/100	53/227	0/93	PF	PF
**TxCF22**	28/421	0/193	39/489	0/186	58/569	0/305	38/664	0/252	114/738	0/174	77/664	0/240	43/654	0/236	208/680	12/319	128/822	3/217	PF	PF
**TxCF23**	4/96	0/82	12/196	0/90	24/42	0/35	2/72	0/25	3/95	0/40	3/129	0/69	4/162	0/28	83/157	0/43	PF	PF	PF	PF

NF = No Fawns; ND = No data; PF = CWD-positive facility.

We used Cap*Ov*CWD to estimate annual CWD detection probabilities for 23 captive cervid facilities in Texas. For 12 of these facilities, data were available for 28–31 years, while the remaining facilities had shorter data histories. Notably, four of the 23 facilities had confirmed CWD detections in 2022 or later. For these CWD-positive facilities, annual CWD detection probabilities were estimated only through the year immediately preceding the first confirmed positive CWD case.

## Results

Overall, CWD detection probabilities estimated using the annual testing data from 2014 to 2020 across the 23 captive cervid facilities were generally low, ranging from 0–56% for adult deer and 0–1% for fawns. Accounting year 2021 onwards, facility-level CWD detection probability estimates rose to 7–88% for adult deer and 0–99% for fawns. However, between 2021 and 2023, only five facilities achieved high detection probabilities (≥ 80%) for adult deer, and only two facilities reached this threshold for fawns (Table 2).

**Table 2:** Annual CWD detection probability estimates for adults (A) and fawns (F) across 23 captive cervid facilities in Texas using accounting year CWD testing data from 2014 to 2023.

Facility ID	2014		2015		2016		2017		2018		2019		2020		2021		2022		2023	
	A	F	A	F	A	F	A	F	A	F	A	F	A	F	A	F	A	F	A	F
**TxCF1**	3	0	22	0	24	0	15	0	6	0	8	0	4	0	40	1	21	0	81	0
**TxCF2**	7	0	8	0	20	0	2	0	12	0	5	0	22	0	25	0	39	0	88	NF
**TxCF3**	5	0	10	0	29	0	4	0	8	0	9	0	12	0	13	0	61	0	88	0
**TxCF4**	6	0	14	0	12	0	22	0	23	0	10	0	13	0	82	32	20	0	68	0
**TxCF5**	0	0	6	0	31	0	8	0	8	0	6	0	8	0	55	15	31	20	80	87
**TxCF6**	ND	ND	ND	ND	50	0	9	0	4	0	3	0	5	0	50	11	30	0	56	0
**TxCF7**	0	NF	0	NF	0	NF	0	NF	0	NF	8	0	6	0	36	8	26	0	24	0
**TxCF8**	1	0	20	0	6	0	2	0	11	0	5	0	8	0	33	21	30	0	57	0
**TxCF9**	4	0	3	0	31	0	4	0	14	0	4	0	13	0	14	46	22	48	30	55
**TxCF10**	0	0	10	0	35	0	6	0	3	0	4	0	3	0	27	0	16	0	53	0
**TxCF11**	1	0	7	0	37	0	3	0	5	0	8	0	13	0	48	4	33	0	48	4
**TxCF12**	5	0	7	0	34	0	5	1	6	0	5	0	7	0	19	0	19	0	27	0
**TxCF13**	0	0	1	0	3	0	1	0	3	0	1	0	2	0	25	0	32	13	27	0
**TxCF14**	ND	ND	0	0	7	0	4	0	8	0	4	0	5	0	33	0	30	0	23	0
**TxCF15**	5	0	5	0	14	0	11	0	8	0	6	0	10	0	49	0	51	2	20	0
**TxCF16**	0	0	3	0	35	0	13	0	3	0	5	0	9	0	12	0	25	0	7	0
**TxCF17**	3	0	2	0	32	0	5	0	5	0	2	0	4	0	70	0	62	99	ND	ND
**TxCF18**	ND	ND	ND	ND	ND	ND	ND	ND	ND	ND	ND	ND	0	0	58	4	28	NF	ND	ND
**TxCF19**	ND	ND	ND	ND	ND	ND	35	1	4	0	8	0	8	0	30	2	ND	ND	ND	ND
**TxCF20**	ND	ND	ND	ND	ND	ND	ND	ND	ND	ND	ND	ND	2	0	48	2	26	1	PF	PF
**TxCF21**	ND	ND	0	0	32	0	5	0	6	0	6	0	7	0	39	0	22	0	PF	PF
**TxCF22**	7	0	8	0	12	0	6	0	19	0	11	0	6	0	32	4	16	1	PF	PF
**TxCF23**	4	0	6	0	56	0	2	0	3	0	2	0	3	0	54	0	PF	PF	PF	PF

Cells shaded in blue indicate high detection probabilities (≥ 80%). NF = No Fawns; ND = No Data; PF = CWD-positive facility. Grey shading indicates facilities with CWD detections.

**Fig 1:**
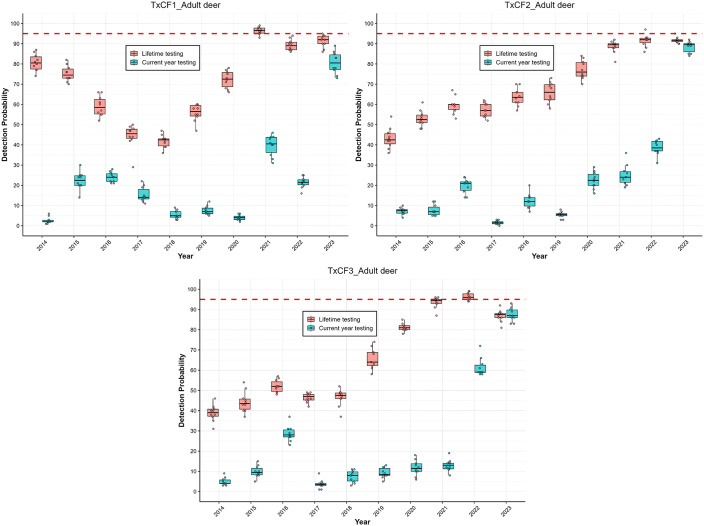
Annual CWD detection probability estimates for adult deer in three captive facilities in Texas. Teal colored boxes indicate detection probabilities based on annual testing data, while red colored boxes represent refined estimates derived from multiyear testing histories (“Lifetime testing”) of individual deer.

Refined CWD detection probability estimates derived from multiyear testing data were consistently higher than those based solely on annual CWD testing data for both adult and fawn deer (Table 3). Using this approach, 12 facilities surpassed the 80% detection probability threshold for adult deer at least once between 2014 and 2023, while 14 facilities surpassed this threshold for fawns during the same period. Notably, three facilities (TxCF1, TxCF2, and TxCF3) maintained high CWD detection probabilities for adult deer across three consecutive years (2021–2023), suggesting a low risk of undetected CWD in their adult populations (Fig 1). Similarly, facility TxCF4 had 100% CWD detection probabilities for both adults and fawns in 2021, with detection probabilities also exceeding 95% for both groups in 2020.

**Table 3:** Annual CWD detection probability estimates for adults (A) and fawns (F) across 23 captive facilities in Texas using multiyear (2014–2025) CWD testing data.

Facility ID	2014		2015		2016		2017		2018		2019		2020		2021		2022		2023	
	A	F	A	F	A	F	A	F	A	F	A	F	A	F	A	F	A	F	A	F
**TxCF1**	81	44	75	36	59	26	44	27	42	76	56	80	72	97	96	95	89	100	91	14
**TxCF2**	43	54	53	39	60	63	57	60	64	55	66	78	77	64	88	71	92	56	92	NF
**TxCF3**	39	64	44	40	52	48	46	33	46	92	66	83	81	44	94	59	96	43	87	2
**TxCF4**	28	43	68	21	56	58	65	40	56	60	76	87	96	100	100	100	50	100	84	32
**TxCF5**	43	35	59	18	54	19	33	18	43	22	60	60	75	56	98	54	62	44	78	88
**TxCF6**	ND	ND	ND	ND	60	0	69	55	53	54	69	64	81	85	90	60	74	79	74	51
**TxCF7**	100	NF	100	NF	100	NF	100	NF	100	NF	28	54	57	54	84	49	57	55	65	34
**TxCF8**	33	21	32	16	18	26	28	32	27	39	26	20	38	54	78	73	64	20	62	0
**TxCF9**	46	13	56	1	56	17	46	4	58	20	59	22	77	17	87	64	81	73	62	57
**TxCF10**	37	46	49	20	44	23	16	57	23	65	41	89	61	69	82	59	65	58	59	7
**TxCF11**	62	67	76	48	73	42	44	46	40	60	53	76	68	75	78	86	65	84	59	69
**TxCF12**	39	30	50	17	43	11	22	30	32	29	37	51	51	87	71	21	68	22	53	0
**TxCF13**	5	17	8	25	14	34	22	42	34	56	47	49	53	58	60	80	56	77	38	18
**TxCF14**	ND	ND	43	44	44	33	37	24	36	16	33	20	42	21	52	48	38	80	32	39
**TxCF15**	42	40	46	50	48	35	49	45	57	61	60	77	71	83	87	45	63	30	35	2
**TxCF16**	56	42	68	30	67	27	47	33	41	47	46	50	53	47	51	40	49	19	32	0
**TxCF17**	38	26	34	28	63	28	49	29	46	38	66	72	89	77	97	42	66	100	ND	ND
**TxCF18**	ND	ND	ND	ND	ND	ND	ND	ND	ND	ND	ND	ND	75	89	81	65	52	NF	ND	ND
**TxCF19**	ND	ND	ND	ND	ND	ND	49	24	34	45	43	76	67	86	57	29	ND	ND	ND	ND
**TxCF20**	ND	ND	ND	ND	ND	ND	ND	ND	ND	ND	ND	ND	55	75	60	21	28	1	PF	PF
**TxCF21**	ND	ND	42	18	43	34	23	26	34	41	42	46	54	26	62	14	24	0	PF	PF
**TxCF22**	53	49	53	35	42	31	34	48	41	65	39	53	42	23	45	16	15	7	PF	PF
**TxCF23**	13	6	14	0	58	12	12	13	11	63	25	32	33	43	58	35	PF	PF	PF	PF

Cells shaded in blue indicate high detection probabilities (≥ 80%). NF = No Fawns; ND = No Data; PF = CWD-positive facility. Grey shading indicates facilities with CWD detections.

For the four facilities that reported CWD detections in 2022 or later, CWD detection probabilities for adults, whether based on annual or multiyear testing data, were consistently below 65%. Although the refined CWD detection probabilities for fawns in these facilities were substantially higher than those estimated using single-year testing data, overall annual CWD detection probabilities remained below 75%. Notably, three of these facilities (TxCF20, TxCF21, TxCF22) had annual CWD detection probabilities for adults consistently below 30% in 2022 (the year before the first confirmed case in these facilities), while the fourth facility (TxCF23) had a CWD detection probability of 58% for adults in 2021 (CWD detected in 2022 in this facility).

## Discussion

CWD detection probability estimates based solely on annual testing data for the 23 captive facilities were low (<56%) for both adult deer and fawns through accounting year 2020. For fawns, detection probabilities were 0 until 2020, except for two facilities where a single fawn was tested in 2017. This reflects the limited CWD testing of fawns, as USDA surveillance requirements mandate testing only for cervids aged 12 months or older [17]. However, when multiyear testing data were incorporated, refined detection probability estimates for fawns increased substantially, offering a more reliable means of assessing the risk of undetected CWD in captive facilities. Beginning in 2021, CWD testing in captive facilities increased following an emergency rule requiring all deer transferred from a facility to a release site to have a CWD not detected test result [26]. In 2023, this mandate was expanded to include deer moved between facilities, further broadening the scope of antemortem testing [27]. As a result, detection probabilities improved for both adult deer and fawns as testing intensity increased. Notably, the multiyear testing data approach revealed even greater improvements in detection probability estimates across years, highlighting its value in assessing the risk of undetected CWD in captive facilities.

Simulation-based approaches, particularly agent-based models, have been employed to investigate CWD dynamics during the early stages of an epidemic and to inform surveillance and management strategies for wild cervid populations [15,16,28–30]. The simulation-based approach using Cap*Ov*CWD described here provides a mechanism for assessing the comprehensive risk of undetected CWD in captive cervid facilities. The annual CWD detection probability estimates provide a measure of confidence in identifying a single CWD-infected deer within a captive cervid herd, based on the number of deer that had a CWD not detected test result throughout the year. Pooling CWD testing data over multiple years for individual deer enables us to refine and optimize CWD detection probability estimates retrospectively. This longitudinal approach accounts for the CWD status of deer that may have been present in a facility during a given year but were not tested for CWD until a later year. CWD testing can occur either in the same facility or another, and may be conducted either antemortem or postmortem, allowing for a more comprehensive understanding of the risk of undetected CWD over time. By incorporating these key factors into the simulation-based approach, the effectiveness of CWD surveillance can be improved, allowing for prioritization of surveillance efforts between captive facilities using the model-derived quantitative metric of detection probability. It is important to note that the simulation-based approach is retrospective in nature as it assesses the effectiveness of past testing to estimate the probability of undetected CWD within a facility, rather than forecasting the risk of future CWD introduction.

If a large proportion of deer in a herd remains untested, the uncertainty about the herd’s true CWD status is amplified. Conversely, as more deer are confirmed CWD-negative—through current-year or multi-year testing—the proportion with uncertain CWD status declines, leading to improved overall CWD detection probability. Facility TxCF2 illustrates this relationship particularly well. Between 2014 and 2024, annual CWD testing of adult deer (both antemortem and postmortem) ranged from 2% to 26% of the herd, with the exception of 2021 and 2023, when testing reached 40% and 80%, respectively. As expected, the annual CWD detection probabilities based solely on testing conducted within a given accounting year remained below 25%, except in the two years with larger sample sizes. In contrast, the refined detection probability estimates—calculated using the cumulative number of confirmed CWD-negative individuals present in the facility during an accounting year—were substantially higher (>40%). Notably, during the three-year period from 2021 to 2023, the refined estimates exceeded 90%, reflecting the impact of intensified sampling and cumulative testing history. This indicates a low likelihood (<10%) of undetected CWD in the facility during those years. Facility TxCF2, along with others that achieved similarly high refined annual CWD detection probabilities (e.g., TxCF3, TxCF4, TxCF5, and TxCF17), may serve as benchmarks for meaningful and sustainable CWD surveillance in captive cervid populations.

Quantifying the likelihood of undetected CWD in a facility provides a valuable metric for evaluating surveillance effectiveness. Notably, all four facilities analyzed in this study that reported CWD detections in 2022 or later had consistently low annual CWD detection probabilities for both adult and fawn deer in the years preceding detection. These low CWD detection probabilities indicate a higher likelihood of undetected CWD in the herd during those years. Low detection probabilities often reflect the current testing guidelines, which allow for lower sampling intensity in facilities characterized by small herd sizes, infrequent transfers, or limited mortality events. Low annual CWD detection probabilities also represent an increased risk of potential exposure and spread of CWD to other facilities. For example, facility TxCF23, which reported 13 positives from 2022 through 2024, had a low CWD detection probability in 2021 (55% current-year, 65% multi-year). Facility TxCF23 had one deer that was later confirmed positive after it had already been transferred from multiple other facilities. Facilities that have not yet detected CWD but exhibit annual CWD detection probabilities below 80% are candidates for enhanced surveillance. For instance, facilities TxCF13, TxCF14, TxCF16, and TxCF19 all had annual CWD detection probabilities for adults below 70%, particularly in the years following 2020. Prioritizing these facilities for increased CWD surveillance efforts could reduce the likelihood of undetected CWD and provide greater confidence that CWD is truly absent from these facilities. A high CWD detection probability does not confirm the absence of disease within a facility unless the CWD detection probability is 100%. However, the value of this approach lies in the metric it provides for comparing surveillance efforts across captive cervid facilities. Importantly, the model also serves as a tool to evaluate and guide the surveillance intensity required to improve facility-level CWD detection probabilities, thereby supporting more informed and effective disease monitoring strategies.

## Management implications

This simulation-based approach offers a practical solution to a key challenge in the disease surveillance of captive cervid facilities: the absence of detected cases in a sampled population does not necessarily indicate disease absence, particularly in low-prevalence scenarios or under-sampled populations. This concern is especially relevant for CWD, where infected animals can shed infectious prions during a prolonged preclinical phase. The resulting environmental contamination makes elimination nearly impossible once the disease becomes established in a facility. Limited sampling may therefore allow the disease to remain undetected within facilities for a period of time before detection, delaying critical containment and management actions. Our approach incorporates heterogeneities in deer demographics, inter-facility movements, and CWD testing efforts, enabling a more nuanced and realistic assessment of surveillance effectiveness. By accounting for herd size, individual deer domiciliary history, and CWD testing records, this approach provides a practical way to interpret “CWD not detected” surveillance results and estimate the likelihood that undetected CWD remains within a facility. Although it cannot guarantee freedom from CWD, it can strengthen confidence when the risk of undetected infection is low. Cap*Ov*CWD can serve as a decision-support tool to standardize surveillance across captive cervid facilities, prioritize facilities for enhanced surveillance, and support more sustainable, efficient, and risk-based CWD monitoring. It can also complement existing surveillance programs by identifying gaps and helping agencies target limited surveillance resources more effectively.

## Supporting information

S1 Text Overview, Design concepts, details (ODD) protocol for Cap*Ov*CWD. (DOCX)

S2 Table Number of adult and fawn deer retrospectively assigned confirmed CWD-negative status based on lifetime CWD testing histories for 23 captive cervid facilities in Texas, 2014–2023. cnA: confirmed negative adult; cnF: confirmed negative fawn; ND = No data; PF = CWD-positive facility. (XLSX)
