# Supplementary material for: A simulation-based approach to strengthen chronic wasting disease surveillance in captive cervid populations
Source: PLoS One. 2026 Jun 24;21(6):e0350825. doi: 10.1371/journal.pone.0350825 (PMC13293428; doi:10.1371/journal.pone.0350825)
Supplement: S1 Text — (DOCX) [file pone.0350825.s001.docx]

**Overview, Design Concepts and Details (ODD) for** **Cap*Ov*CWD**

<https://doi.org/10.25937/87rx-af75>

**Background**

The spread of infectious diseases in captive facilities, such as breeding pens or high-fence enclosures, can be rapid and difficult to control. Chronic wasting disease (CWD) is a progressive, invariably fatal, neurodegenerative disease of cervids that is transmitted both through direct contact between animals and indirect contact via contaminated environmental fomites. CWD circulates in both wild and captive cervid populations across North America. In captive facilities such as breeding pens or high-fence enclosures, the risk of rapid spread is particularly high due to elevated animal densities and shared environments. Once introduced, chronic wasting disease (CWD) often spreads quickly within a facility, leading to high infection rates and elevated prevalence levels. This underscores the need for proactive surveillance approaches that can detect CWD early—before it becomes established—so that timely interventions can be implemented to reduce the risk of persistence in captive herds and spillover into surrounding wild cervid populations. To address this challenge, we developed a simulation-based framework for estimating facility-level CWD detection probabilities. Our agent-based model, **Cap*Ov*CWD**, simulates the sampling process within captive herds and calculates detection probabilities using both current-year sampling data and multi-year testing histories, providing a more comprehensive assessment of the likelihood of undetected CWD.

**Purpose**

**Cap*Ov*CWD** is an agent-based model that simulates a captive cervid herd composed of adults and fawns. The model deer population is initialized using data on herd size and composition from captive facility records. Individual deer domiciliary history and annual CWD testing records inform the herd size and sample size (for CWD testing), respectively. The model can be used to iteratively estimate the facility level annual CWD detection probability. Detection probability estimates can be further refined by incorporating multiyear CWD testing data. This approach can be particularly useful for interpreting negative test results from a subset of the captive herd. Facility level detection probability estimates provide a comprehensive and standardized risk metric that reflects the likelihood of undetected CWD in the facility.

**Entities, State Variables and Scales:**

*Entities* **Cap*Ov*CWD** has two entities: adult deer and fawn deer. Each deer is represented as an individual agent. Deer are classified as either an adult or a fawn, with age class assigned based on the midpoint of the accounting year, when fawns from the previous year transition to the adult stage. The only state variable for adult and fawn deer is CWD status (0 = uninfected, 1 = infected).

*Spatial scale*: The model is not spatially explicit; the model landscape just represents a captive deer facility.

*Temporal scale*: **Cap*Ov*CWD** runs for 1 time step during which the population snapshot for the captive herd is sampled for CWD testing.

**Process overview and scheduling**

The model runs in NetLogo. One or more deer from respective age class are randomly designated as CWD-infected (num_inf_adults, num_inf_fawns). Sampling is then simulated from the defined testing pool, with the sample size specified either as the number of deer tested for CWD in the current year (tested_adults; tested_fawns) or as the total number of confirmed CWD-negative deer within the facility based on lifetime testing history (neg_adults; neg_fawns). If at least one CWD-infected deer is included in the tested subset, the detection probability is recorded as 1; otherwise, it is 0. For each iteration, detection probability is estimated separately for adults and fawns. Within each age class, detection probability is calculated using two sample size definitions: (i) the number of individuals tested in the current year, and (ii) the number of deer confirmed CWD-negative in the given year based on lifetime testing. Using an iterative approach, (e.g., 1,000 iterations), annual CWD detection probability estimates for a captive facility can be determined as the proportion of iterations in which the CWD-positive individual was included in the sample of tested deer.

**Design Concepts**

*Emergence*: The emergent effect of interest here is the detection (or non-detection) of an infected agent in the agent population if a subset of agents are randomly selected.

*Stochasticity*: A subset of agents are selected stochastically per each iteration..

*Observation*: The inclusion of infected agent in the subset (sample) of agents is documented.

**Initialization**

Model population is initialized based on user-provided input (year, num_adults, num_fawns) to represent the captive herd in a selected accounting year. An accounting year spans from April 1 to March 31 of the following year. Deer that were present in the facility at any point during an accounting year are included in that year’s model population, including individuals that died or were transferred in or out before the end of the year. These deer are included in the model to represent a “worst case scenario” for facilities to estimate the risk of undetected CWD in the facility. Age class is assigned based on the midpoint of the accounting year, when fawns from the previous year transition to the adult stage.

Testing data for a captive facility, both ante-mortem and postmortem tests conducted within a selected year, informs the number of adult and fawn deer tested during each accounting year (tested_adults; tested_fawns). Negative test results for individual deer from the current or subsequent years inform the number of confirmed CWD-negative deer in a given year (neg_adults; neg_fawns).

**Input data**

The model does not use input data to represent time-varying processes.

**Submodels**

No submodels.
