# Supplementary material for: A simulation-based approach to strengthen chronic wasting disease surveillance in captive cervid populations
Source: PLoS One. 2026 Jun 24;21(6):e0350825. doi: 10.1371/journal.pone.0350825 (PMC13293428; doi:10.1371/journal.pone.0350825)
Supplement: Attachment — Submitted filename: Response_to_reviewers.docx [file pone.0350825.s004.docx]

**Response to reviewers**

We are grateful to the reviewers and the editor for their comments and suggestions. Along with the revised manuscript, we provide this detailed response to editor and reviewer comments. Our responses to reviewer comments are in red. Lines from the manuscript, or changes in response to the comments in the revised manuscript are in blue.

**Reviewer #1**: Wakefield et al. “A simulation-based approach to strengthen chronic wasting disease surveillance in captive cervid populations”

The authors have taken historical data from the US state of Texas and performed simulations to determine the likelihood of having detected CWD in negative farms. This tool can be useful for identifying facilities that should increase testing so that they can be confident that negative results in a subset of animals are more likely to be reflective of a CWD negative facility. It remains unclear, however, whether this information could be gleaned from simply looking at the proportion of animals tested in each facility.

Response: Thank you for the thorough review. We have addressed your concerns and incorporated most of the suggestions.

Major points:
The requirements of the USDA herd certification program should be clearly stated. It is my understanding that while there is no requirement that a percentage of the heard be tested in a given year, in addition to animals that die at an age greater than 12 months, any animal greater than 12 months old slaughtered on or off site must also be tested.

Response: USDA herd certification program requirements are discussed here:

Lines 78: A key requirement of this program is that all on-farm deaths of cervids aged 12 months or older must be tested for CWD. Five consecutive years of CWD-free testing is required to certify herds as being low risk for having CWD, and animals from such herds may be shipped interstate.

Also, line 86: Individual states may implement regulations that are more stringent than the HCP requirements. For example, in Texas, in addition to requiring CWD testing of all mortalities within captive cervid facilities, ante-mortem testing is also mandated for any deer being moved from a facility.

On a farm, one would expect this to be a large number of the heard, or at the very least a relatively consistent percentage from harvest to harvest.

Response: The number of animals tested in captive cervid facilities is generally low (see new table 1). Under the USDA Herd Certification Program, only on-farm mortalities of cervids older than 12 months are required to be tested for CWD, and in Texas, ante-mortem testing is additionally required for deer moved out of a facility. Consequently, only a subset of the herd is tested in any given year. In facilities with low mortality, small herd size, or limited animal movement, the number of deer tested may be very small and, in some years, no deer may be tested at all.

This leads into my second point: it would benefit the reader to have a summary of the real-life data associated with each facility in Tables 1 and 2. How many animals were in these facilities in each year, and how many/what proportion were tested?

Response: New table 1 added: proportion of deer tested

What tests were used? What was the sensitivity of these tests? Without this information, it is difficult to assess the benefit of this modeling. Are these detection probabilities simply a reflection of the proportion of animals tested?

Response: Line 147: In addition to the required post-mortem testing of retropharyngeal lymph nodes and brainstem tissue using IHC or ELISA, TPWD and the Texas Animal Health Commission (TAHC) have incorporated ante-mortem testing using immunohistochemistry (IHC) of samples obtained via tonsil biopsy or rectal biopsy.

The number of deer tested in the captive facilities may be very small and, in some years, no deer may be tested at all (See new Table 1). We estimate the facility-level probability of detecting a single infected deer given the sampling intensity achieved and the diagnostic tests currently in use.

It is unclear from the methodology exactly how animal movement from one facility to another is taken into account. Movement would generally be assumed to entail additional risk to the facility, is this incorporated into the model? Or is this Texas specific, where animals that move must first be tested?

Response: Animal movement between facilities is incorporated through the individual deer transfer histories used to initialize the model for each accounting year. For each facility, the Texas Parks and Wildlife Department maintains annual records of herd size, herd composition, transfers into and out of the facility, and individual deer testing histories. These data are used to construct the model population for each accounting year.

Any deer that was present in a facility at any point during a given accounting year is included in that year’s model population, even if the animal was later transferred or died before the end of the year. Thus, deer moved into a facility contribute to the population at risk during the year they were present, and deer moved out remain part of the facility’s annual risk profile for that year.

We intentionally adopted this approach to represent a conservative, “worst-case” scenario for estimating the likelihood of undetected CWD. Because an infected deer may have been present in the facility for only part of the year before being transferred or dying, excluding such individuals could underestimate the facility-level risk of undetected CWD.

See lines145-149: Deer that were present in the facility at any point during an accounting year are included in that year’s model population, including individuals that died or were transferred in or out before the end of the year. These deer are included in the model to represent a “worst case scenario” for facilities to estimate the risk of undetected CWD in the facility.

Figure 1: What do the individual data points represent?

Response: The individual data points represent annual CWD detection probabilities (y-axis) for a given accounting year (x-axis). Annual detection probability is estimated as the proportion of model iterations in which CWD-infected deer is included in the tested sample, thereby resulting in successful detection of CWD within the facility. For each accounting year, 1000 iterations were conducted and summarized into 10 replicate detection probability estimates (each based on 100 iterations), which are shown as the individual data points.

Line 172 changed: Annual CWD detection probability was estimated as the proportion of iterations in which the CWD-infected deer was included in the tested sample, thereby resulting in successful detection of CWD.

Line 191: For each accounting year, 1000 iterations were conducted and summarized into 10 replicate detection probability estimates (each based on 100 iterations). 

The statement on line 85: "Even with the requirement for anti-mortem testing of all cervids prior to movement from a facility, sample sizes are often insufficient to confidently detect CWD in low prevalence scenarios". This requirement, presumably, refers to the additional mandates from the state of Texas. However, if all animals are tested, how could the sample size be insufficient? Please clarify this statement.

Response: All animals in a facility are not tested - under the USDA Herd Certification Program, only on-farm mortalities of cervids older than 12 months are required to be tested for CWD, and in Texas, ante-mortem testing is additionally required for deer moved out of a facility. Consequently, only a subset of the herd is tested in any given year. In facilities with low mortality, small herd size, or limited animal movement, the number of deer tested may be very small and, in some years, no deer may be tested at all. Even when all required animals test “CWD not detected,” these animals may represent only a small proportion of the herd. The central question addressed in this paper is what such negative results imply about the likelihood that undetected CWD may still be present in the remainder of the facility.

Editorial points:
Line 36: “misfolded isoforms” should be changed, as prions are not isoforms of the prion protein. Suggestion of “misfolded forms” or “misfolding of the normal..”

Response: Changed to ‘..misfolded forms of the normal host cellular prion protein.’

Line 39: CWD has also been detected in Europe and Asia

Response: Added the following: CWD has also been detected in free-ranging reindeer (*Rangifer tarandus*) in Norway and in captive cervids in South Korea. Moreover, a novel form of CWD with atypical characteristics has been reported in moose and red deer in Norway, as well as in moose in Finland and Sweden (Pirisinu et al. 2018, Vikøren et al. 2019, Mysterud et al. 2020, Hopp et al. 2024). These cases appear to represent sporadic CWD (sCWD), with little or no evidence of transmission between live animals.

Hopp P, Rolandsen CM, Korpenfelt S-L, Våge J, Sörén K, Solberg EJ, Averhed G, Pusenius J, Rosendal T, Ericsson G, Bakka HC, Mysterud A, Gavier-Widén D, Hautaniemi M, Ågren E, Isomursu M, Madslien K, Benestad SL, Nöremark M. 2024. Sporadic cases of chronic wasting disease in old moose – an epidemiological study. Journal of General Virology 105.

Mysterud A, Benestad SL, Rolandsen CM, Våge J. 2020. Policy implications of an expanded chronic wasting disease universe. Journal of Applied Ecology 58: 281–285.

Pirisinu L, Tran L, Chiappini B, Vanni I, Di Bari MA, Vaccari G, Vikøren T, Madslien KI, Våge J, Spraker T, Mitchell G, Balachandran A, Baron T, Casalone C, Rolandsen CM, Røed KH, Agrimi U, Nonno R, Benestad SL. 2018. Novel Type of Chronic Wasting Disease Detected in Moose (*Alces alces*), Norway. Emerging Infectious Diseases 24: 2210–2218.

Vikøren T, Våge J, Madslien KI, Røed KH, Rolandsen CM, Tran L, Hopp P, Veiberg V, Heum M, Moldal T, Neves CGD, Handeland K, Ytrehus B, Kolbjørnsen Ø, Wisløff H, Terland R, Saure B, Dessen KM, Svendsen SG, Nordvik BS, Benestad SL. 2019. First Detection of Chronic Wasting Disease in a Wild Red Deer (*Cervus elaphus*) in Europe. Journal of Wildlife Diseases 55: 970.

Line 47: suggest “the US state of South Dakota”

Response: Changed to ‘..from the US state of South Dakota..’

Line 48: suggest “the Canadian province of Saskatchewan”

Response: Changed to ‘…the Canadian province of Saskatchewan…’

Line 51: “fully impermeable” suggest “impermeable”

Response: Changed to ‘Moreover, there is a persistent risk of reciprocal CWD transmission between captive and wild cervids, because fences are permeable (Gerhold & Hickling, 2016).’

Line 54: “prevalence levels” suggest “prevalence”

Response: Changed to ‘..and in some cases, prevalence nearing 100%’

Line 57: “infectious prions” suggest “prions”

Response: Contamination is due to the prions shed by the CWD+ deer that are, and remain infectious in the environment, hence infectious prions.

Line 59: “infection” suggest “infectivity”

Response: Changed to ‘..creating reservoirs of infectivity..’

Line 61: suggest reference for the environmental persistence of prions
-Johnson, Christopher J., et al. "Prions adhere to soil minerals and remain infectious." PLoS pathogens 2.4 (2006): e32.

Response: Reference added: Johnson CJ, Phillips KE, Schramm PT, McKenzie D, Aiken JM, Pedersen JA. 2006. Prions adhere to soil minerals and remain infectious. PLoS Pathogens 2: e32.

Line 289: “infectious prions” suggest “prions”

Response: The prions shed by the CWD+ deer are, and remain infectious in the environment, hence infectious prions.

**Reviewer #2**: There are two major limitations to this approach. Firstly, cervids do not exhibit positivity in peripheral tissues like the tonsil until 6-18 months post exposure (see Mathiason et al. 2009 and others). This sensitivity issue is compounded when only IHC is used instead of sensitive amplification assays (e.g. real-time quaking induced conversion). This delay to positivity was not accounted for in the simulation, and further, the authors claim a 99% negative predictive value. This is simply impossible with a disease like chronic wasting disease that can take years to manifest.

Response: The reviewer’s point regarding delay to positivity is logical, but it does not directly apply to the purpose of this analysis. We are not using this approach to certify that a facility is free of CWD. Rather, we estimate the facility-level probability of detecting a single infected deer given the sampling intensity achieved and the diagnostic tests currently in use. Thus, the approach is designed to evaluate the confidence that can be placed in existing surveillance rather than to declare disease freedom.

Our approach provides a practical means of interpreting “CWD not detected” results from a subset of the herd by accounting for herd size, individual deer domiciliary history, and CWD testing records. In doing so, it offers regulatory agencies a decision-support tool for standardizing surveillance across captive cervid facilities and promoting more sustainable and efficient CWD surveillance strategies.

Also, the comment that a 99% negative predictive value (NPV) is impossible is not correct. Unlike diagnostic sensitivity, which is an intrinsic property of a test, NPV depends strongly on the pre-test probability of disease and therefore increases as disease prevalence decreases. In low-prevalence situations such as those considered here (<1%), even diagnostic tests with relatively modest sensitivity (e.g., 25–70%) can have NPVs exceeding 99%, because the large majority of animals tested are truly uninfected. This relationship is well established in diagnostic epidemiology; see, for example, Monaghan et al. 2021. [10.3390/medicina57050503](https://doi.org/10.3390/medicina57050503)

Secondly, the percentage of the herd tested needs to be accounted for in the model. The authors mention the importance of this consideration, yet it was unclear if the percentage of herd tested was accounted for in the model.

Response: Yes, the proportion of the herd tested is indeed accounted for in this agent-based model. Please refer to line 133: Cap*Ov*CWD was initialized using data on herd size, composition, and individual deer transfer histories obtained from captive facility records maintained by the Texas Parks and Wildlife Department (TPWD).

Line 145: The number of adult and fawn deer tested for CWD in each accounting year was determined from individual testing histories, which included both ante-mortem and post-mortem test results recorded within the accounting year.

Minor comments include the following: authors should consider biological sex as a variable for chronic wasting disease modeling, and figure legend specifies green when the color in the figure appears blue.

Response: This approach does not really model chronic wasting disease, we are just simulating CWD surveillance scenarios in captive facilities to determine facility-level CWD detection probabilities. Biological sex can be readily incorporated in the model for estimating CWD detection probabilities.

Changed figure caption: Teal colored boxes indicate detection probabilities based on annual testing data, while red colored boxes represent refined estimates derived from multiyear testing histories (“Lifetime testing”) of individual deer.

**Reviewer #3**: The manuscript by Wakefield, L. et al (PONE-D-25-67529) entitled, “A simulation-based approach to strengthen chronic wasting disease surveillance in captive cervid populations,” explains the authors’ simulation-based method to analyze CWD surveillance results with the purpose to support monitoring of cervid herds. The study is straightforward and the manuscript is well written. Overall the approach may prove to be useful for CWD surveillance policy. While enthusiasm for the manuscript is high, there are a few concerns the authors should address.

Lines 240-243: As the authors note here, the approach is retrospective in nature. Such a point should be highlighted and discussed throughout the manuscript, beginning in the abstract. The abstract does not clearly state how the approach should be used, but rather summarizes that the approach addresses the issue with negative CWD results. Instead the authors should state early on that this is a retrospective approach that can be applied to historical CWD testing data and be used to identify at-risk herds. Throughout the manuscript, especially the management implementation section, the authors should highlight the application of the approach to identify at risk herds. This is a valuable application of the approach and should be a focus of the authors descript of the use of the application.

Response: Following changes made as per the suggestions:

Abstract, line 27: This simulation-based approach is retrospective in nature. By integrating deer demographic data, individual movement histories, and past CWD testing records, this tool estimates facility-level CWD detection probabilities, providing a clearer assessment of the likelihood of undetected disease within a facility.

Introduction, line 106: By applying the model to historical CWD testing data, facilities with a higher likelihood of undetected CWD can be identified, even when CWD is not detected in the subset of animals tested.

Management implications, line 313: By accounting for herd size, individual deer domiciliary history, and CWD testing records, this approach provides a practical way to interpret “CWD not detected” surveillance results and estimate the likelihood that undetected CWD remains within a facility. Although it cannot guarantee freedom from CWD, it can strengthen confidence when the risk of undetected infection is low. Cap*Ov*CWD can serve as a decision-support tool to standardize surveillance across captive cervid facilities, prioritize facilities for enhanced surveillance, and support more sustainable, efficient, and risk-based CWD monitoring. It can also complement existing surveillance programs by identifying gaps and helping agencies target limited surveillance resources more effectively.

Lines 85-87: The authors write, “…sample sizes are often insufficient to confidently detect CWD in low-prevalence scenarios,” but can the authors provide a literature citation to statistical data that support this statement? Have there been any studies that performed power analyses, false discovery rate (q-value), or other statistical analyses to determine the sample size needed for confident determination of CWD presence or absence for a given testing method?

Response: Literature citation provided.

Sample sizes are often insufficient to confidently detect CWD in such low-prevalence scenarios (Belsare et al. 2020, 2021).

The introduction/background section would benefit from a brief discussion of the CWD testing methods and their sensitivity, as mentioned in the section of Lines 122-141. For example, the RT-QulC test on deer urine other biological samples, like blood or cerebrospinal fluid. Such a discussion would help put the shortcomings of testing in context for the manuscript.

Response: Added in Introduction, line 90: CWD diagnosis primarily relies on postmortem detection of abnormal prion proteins in retropharyngeal lymph nodes and brainstem tissue using immunohistochemistry (IHC), enzyme-linked immunosorbent assay (ELISA), or western blotting, although approved routine diagnostic testing is currently limited to IHC and ELISA (USDA APHIS 2019). Ante-mortem testing of peripheral lymphoid tissues, particularly tonsil and recto-anal mucosa-associated lymphoid tissue (RAMALT) by immunohistochemistry can improve the probability of detecting CWD infected animals before death, especially during the early stages of infection (Thomsen et al. 2012, Monello et al. 2013, Schneider et al. 2023). More recently, amplification assays such as protein misfolding cyclic amplification (PMCA) and real-time quaking-induced conversion (RT-QuIC) have demonstrated substantially greater sensitivity than conventional diagnostic methods, allowing detection of very low concentrations of prions in tissues and body fluids (Haley et al. 2016). These approaches may permit earlier detection of CWD during the preclinical phase of infection and from alternative sample types collected before or after death, thereby improving the likelihood of detecting infected animals when prevalence is low or infection is recent.

Lines 50-51: Can the authors state this in the positive? For example, “There is a persistent risk of reciprocal CWD transmission between captive and wild cervids, because fences are permeable.”

Response: Changed: Moreover, there is a persistent risk of reciprocal CWD transmission between captive and wild cervids, because fences are permeable (Gerhold & Hickling, 2016).

Lines 142-161: A salient point that the authors should explicitly state is whether testing scenarios re-tested previously tested deer and what proportion of the annual sample test size (e.g. was 1 out of 10 deer previously tested?).

Response: For each accounting year, we simulated two scenarios per age class (adults and fawns) in each captive facility. For the first scenario, sample size was defined as the number of deer in the captive facility that were tested during an accounting year and had “CWD not detected” results, based on TPWD records. During each model iteration, one deer from the relevant age class was randomly designated as CWD-infected, and a sample of the specified size was then randomly selected for CWD testing. Annual CWD detection probability was estimated as the proportion of iterations in which the CWD-infected deer was included in the tested sample, thereby resulting in successful detection of CWD.

Additionally, the authors state that 1,000 iterations were tested. How was each iteration varied between one another? Was a new iteration a random selection, semi-random selection, or non-random selection of tested deer?

Response: Cap*Ov*CWD was developed in NetLogo, a software platform widely used for implementing agent-based models. Randomness is an integral component of NetLogo simulations and is used throughout the model to represent stochastic real-world processes, including the selection of infected deer and the random selection of deer for CWD testing.

Finally, how were sampling sizes determined? Is a percentage of the heard tested? It would be helpful if there was some statistical analysis to help determine the testing sample size given the population size (and possibly including the variable of pen size- like 1 sampled deer per 10 deer per acre). If such parameters are already accounted for in CapOvCWD-then please reference these points in this section.

Response: Line 168: For the first scenario, sample size was defined as the number of deer in the captive facility that were tested during an accounting year and had “CWD not detected” results, based on TPWD records.

Line 176: For the second scenario, the total number of confirmed CWD-negative individuals present in the facility during the accounting year, derived from multiyear testing data, was used as the sample size to refine the annual CWD detection probability estimates.
