# Supplementary material for: A simulation-based approach to strengthen chronic wasting disease surveillance in captive cervid populations
Source: PLoS One. 2026 Jun 24;21(6):e0350825. doi: 10.1371/journal.pone.0350825 (PMC13293428; doi:10.1371/journal.pone.0350825)
Supplement: Attachment — Submitted filename: Response to Reviewers.docx [file pone.0350825.s005.docx]

**05/18/2026**

**Response to reviewers**

We are grateful to the reviewers and the editor for their comments and suggestions. Along with the revised manuscript, we provide this detailed response to reviewer comments. Our responses to reviewer comments are in blue. Changes in response to the comments in the revised manuscript are in red.

**Reviewer #1:** The authors have responded adequately to my initial critiques. My suggestion that “infectious prions” be changed to “prions” is rooted in the fact that prions are, by definition, infectious, making the statement “infectious prions” redundant. I will not belabour this point further, however.

**Response**: Line 63: Infectious prions changed to prions.

---

The inclusion of the new table 1, which provides the proportion of animals tested in each year, reiterates a point made in the first round of review. At first glance, it appears that the model is simply assigning confidence to years in which a very large proportion of animals were tested. For example, in 2023 TxCF1-4 had the following proportions of animals tested: 208/260, 237/265, 423/477, 41/61, 227/288. Looking at these numbers alone, I would conclude that it is unlikely that CWD was present in these facilities, and it is unclear to me what additional insight is provided by this modeling in these scenarios.

**Response**: The annual CWD detection probabilities estimated using the proportion of animals tested every year (Table 1) are presented in Table 2. **Additional insights** provided by the modeling approach are in **Table 3.** For the 5 facilities highlighted by Reviewer 1 (TxCF1-5), note that increased testing resulted in increased CWD detection probability (> 80%) only in the year when more deer were tested (2023 for TxCF1,2,3,5 and 2021 for TxCF4) - see Table 2. But for the same data, our modeling approach provides greater improvements in detection probability estimates across years – see table 3.

Please check line270: Facility TxCF2 illustrates this relationship particularly well. Between 2014 and 2024, annual CWD testing of adult deer (both antemortem and postmortem) ranged from 2% to 26% of the herd, with the exception of 2021 and 2023, when testing reached 40% and 80%, respectively. As expected, the annual CWD detection probabilities based solely on testing conducted within a given accounting year remained below 25%, except in the two years with larger sample sizes. In contrast, the refined detection probability estimates—calculated using the cumulative number of confirmed CWD-negative individuals present in the facility during an accounting year—were substantially higher (>40%). Notably, during the three-year period from 2021 to 2023, the refined estimates exceeded 90%, reflecting the impact of intensified sampling and cumulative testing history. This indicates a low likelihood (<10%) of undetected CWD in the facility during those years. Facility TxCF2, along with others that achieved similarly high refined annual CWD detection probabilities (e.g., TxCF3, TxCF4, TxCF5, and TxCF17), may serve as benchmarks for meaningful and sustainable CWD surveillance in captive cervid populations.

---

In the case of TxCF18-19, there is high confidence that fawns in 2020 did not have CWD despite no fawns being tested.

Response: Exactly, the modeling approach provides high confidence despite low/no testing - this is explained in the manuscript:

Line 255: Pooling CWD testing data over multiple years for individual deer enables us to refine and optimize CWD detection probability estimates retrospectively. This longitudinal approach accounts for the CWD status of deer that may have been present in a facility during a given year but were not tested for CWD until a later year. CWD testing can occur either in the same facility or another, and may be conducted either antemortem or postmortem, allowing for a more comprehensive understanding of the risk of undetected CWD over time.

---

In other instances in 2023, the confidence is low using multi-year data despite relatively high testing percentages (TxCF5, 11, 13). I feel the manuscript would be improved if this, and potentially other examples, are explicitly discussed in this light to further highlight the usefulness of this modeling.

**Response**: Please also check line 297: For instance, facilities TxCF13, TxCF14, TxCF16, and TxCF19 all had annual CWD detection probabilities for adults below 70%, particularly in the years following 2020. Prioritizing these facilities for increased CWD surveillance efforts could reduce the likelihood of undetected CWD and provide greater confidence that CWD is truly absent from these facilities.

----

**Reviewer #2**:

As the authors point out, a 99% negative predictive value can be achieved from low prevalence scenarios. However, the current verbiage is "Across this sensitivity range, the NPV exceeded 99%, indicating a high likelihood that deer with CWD not detected results using ante-mortem methods were free of CWD, supporting the inclusion of CWD not detected ante-mortem tests as reliable indicators of CWD-negative status in the model.” Change to “Across this sensitivity range and prevalence <1%, the NPV exceeded 99%, indicating a high likelihood that deer with CWD not detected results using ante-mortem methods were free of CWD, supporting the inclusion of CWD not detected ante-mortem tests as reliable indicators of CWD-negative status in the model. However, NPV will decrease as prevalence increases, and as such, the 99% NPV value is only applicable for low prevalence scenarios” or something similar. This should be abundantly clear in the manuscript.

**Response**: Line 152 changed as follows:

Across this sensitivity range under a low prevalence scenario, NPV exceeded 99%, indicating a high likelihood that deer with CWD not detected ante-mortem test results were free of CWD within the limitations of currently available diagnostics. This supports the use of ante-mortem CWD not detected results as reliable indicators of CWD-negative status within the model framework. Although NPV declines at higher prevalence levels when test sensitivity is low, falling below 90% when prevalence exceeds 10%, surveillance at such prevalence levels requires substantially smaller sample sizes for confident detection of CWD, reducing the practical need for this modeling approach.

---
At a minimum, authors should mention the unique aspects of CWD pathogenesis that lead to delays in positivity, even for antemortem samples. Their simulation relies on gold standard diagnostic tests that require months post exposure to show positivity. This must be mentioned in the manuscript as a limitation to data interpretation. Specifically, a negative test result could be interpreted as the animal is truly CWD-free or the animal has been exposed but has not reached the threshold to positivity.

**Response**: Added the following: Line 101: At present, no diagnostic test can reliably determine that an individual animal is free of CWD. Existing tests are not sufficiently sensitive to detect all infected animals, particularly during the early stages of infection, because CWD has a prolonged incubation period that can result in false-negative test outcomes before prion accumulation reaches detectable levels.

---

For example, "... a CWD not detected test result indicates that the deer was not infected in any previous year" is simply not accurate.

**Response**: Changed as follows: Line 171: Therefore, within the limitations of currently available diagnostics, a CWD not detected test result indicates a high likelihood that the deer was not infected in previous years.

---

In the response to reviewer comments, the authors mention “…the approach is designed to evaluate the confidence that can be placed in existing surveillance rather than to declare disease freedom.” As written, the manuscript does not reflect this. For example, "... a high likelihood that deer with CWD not detected results using ante-mortem methods were free of CWD, supporting the inclusion of CWD not detected ante-mortem test results as reliable indicators of CWD-negative status in the model." Further, "..CapOvCWD described here provides a mechanism for assessing the comprehensive risk of undetected CWD in captive cervid facilities."

**Response**: Changed as above:

Line 152: Across this sensitivity range under a low prevalence scenario, NPV exceeded 99%, indicating a high likelihood that deer with CWD not detected ante-mortem test results were free of CWD within the limitations of currently available diagnostics. This supports the use of ante-mortem CWD not detected results as reliable indicators of CWD-negative status within the model framework.

Please consider this as well:

Line 332: Cap*Ov*CWD can serve as a decision-support tool to standardize surveillance across captive cervid facilities, prioritize facilities for enhanced surveillance, and support more sustainable, efficient, and risk-based CWD monitoring. It can also complement existing surveillance programs by identifying gaps and helping agencies target limited surveillance resources more effectively.
---
Lastly, as presented, it is still unclear whether the percentage of herd tested was explicitly included in the simulation. The authors refer to two separate lines that point out these variables, but it would be helpful to make this more concise.

**Response**: Cap*Ov*CWD (the model described in this paper) is an agent-based simulation model. The model initializes a captive deer population (the denominator) and simulates testing of a subset of this population (numerator).

To clarify, the herd (denominator) is explained in line 134: Deer that were present in the facility at any point during an accounting year are included in that year’s model population, including individuals that died or were transferred in or out before the end of the year.

The numerator (sample size or the subset of this population tested for CWD) is explained in line 141: The number of adult and fawn deer tested for CWD in each accounting year was determined from individual testing histories, which included both ante-mortem and post-mortem test results recorded within the accounting year.

Line 165: sample size was defined as the number of deer in the captive facility that were tested during an accounting year and had “CWD not detected” results,

Line 167: During each model iteration, one deer from the relevant age class was randomly designated as CWD-infected, and a sample of the specified size was then randomly selected for CWD testing.
