# Supplementary material for: A simulation-based approach to strengthen chronic wasting disease surveillance in captive cervid populations
Source: PLoS One. 2026 Jun 24;21(6):e0350825. doi: 10.1371/journal.pone.0350825 (PMC13293428; doi:10.1371/journal.pone.0350825)
Supplement: Peer review history

# Peer review: Decision Letter 0

**DOI:** 10.1371/journal.pone.0350825.r001

**Authors:** Jung-Yong Yeh

13 Apr 2026

PONE-D-25-67529

A simulation-based approach to strengthen chronic wasting disease surveillance in captive cervid populations

PLOS One

Dear Dr. Belsare,

Thank you for submitting your manuscript to PLOS ONE. After careful consideration, we feel that it has merit but does not fully meet PLOS ONE’s publication criteria as it currently stands. Therefore, we invite you to submit a revised version of the manuscript that addresses the points raised during the review process.

Please submit your revised manuscript by May 28 2026 11:59PM. If you will need more time than this to complete your revisions, please reply to this message or contact the journal office at plosone@plos.org. When you're ready to submit your revision, log on to https://www.editorialmanager.com/pone/ and select the 'Submissions Needing Revision' folder to locate your manuscript file.

Please include the following items when submitting your revised manuscript:

- A letter that responds to each point raised by the academic editor and reviewer(s). You should upload this letter as a separate file labeled 'Response to Reviewers'.
- A marked-up copy of your manuscript that highlights changes made to the original version. You should upload this as a separate file labeled 'Revised Manuscript with Track Changes'.
- An unmarked version of your revised paper without tracked changes. You should upload this as a separate file labeled 'Manuscript'.

If you would like to make changes to your financial disclosure, please include your updated statement in your cover letter. Guidelines for resubmitting your figure files are available below the reviewer comments at the end of this letter.

If applicable, we recommend that you deposit your laboratory protocols in protocols.io to enhance the reproducibility of your results. Protocols.io assigns your protocol its own identifier (DOI) so that it can be cited independently in the future. For instructions see: https://journals.plos.org/plosone/s/submission-guidelines#loc-laboratory-protocols. Additionally, PLOS ONE offers an option for publishing peer-reviewed Lab Protocol articles, which describe protocols hosted on protocols.io. Read more information on sharing protocols at https://plos.org/protocols?utm_medium=editorial-email&utm_source=authorletters&utm_campaign=protocols.

As the corresponding author, your ORCID iD is verified in the submission system and will appear in the published article. PLOS supports the use of ORCID, and we encourage all coauthors to register for an ORCID iD and use it as well. Please encourage your coauthors to verify their ORCID iD within the submission system before final acceptance, as unverified ORCID iDs will not appear in the published article. *Only* the individual author can complete the verification step; PLOS staff *cannot* verify ORCID iDs on behalf of authors.

We look forward to receiving your revised manuscript.

Kind regards,

Jung-Yong Yeh

Academic Editor

PLOS One

Journal Requirements:

When submitting your revision, we need you to address these additional requirements.

1. Please ensure that your manuscript meets PLOS ONE's style requirements, including those for file naming. The PLOS ONE style templates can be found at

https://journals.plos.org/plosone/s/file?id=wjVg/PLOSOne_formatting_sample_main_body.pdf and

https://journals.plos.org/plosone/s/file?id=ba62/PLOSOne_formatting_sample_title_authors_affiliations.pdf.

2. Thank you for stating in your Funding Statement:

" This work was supported by the United States Department of Agriculture, Animal and Plant Health Inspection Service (USDA APHIS) under grant number AP23VSSPRS00C130. LW was also supported through funding from a Safari Club International Foundation grant. The funders had no role in the study design, data collection and analysis, decision to publish, or preparation of the manuscript."

Please provide an amended statement that declares *all* the funding or sources of support (whether external or internal to your organization) received during this study, as detailed online in our guide for authors at http://journals.plos.org/plosone/s/submit-now. Please also include the statement “There was no additional external funding received for this study.” in your updated Funding Statement.

Please include your amended Funding Statement within your cover letter. We will change the online submission form on your behalf.

3. We note that your Data Availability Statement is currently as follows: [All relevant data are within the manuscript and its Supporting Information files.]

Please confirm at this time whether or not your submission contains all raw data required to replicate the results of your study. Authors must share the “minimal data set” for their submission. PLOS defines the minimal data set to consist of the data required to replicate all study findings reported in the article, as well as related metadata and methods (https://journals.plos.org/plosone/s/data-availability#loc-minimal-data-set-definition).

For example, authors should submit the following data:

- The values behind the means, standard deviations and other measures reported;

- The values used to build graphs;

- The points extracted from images for analysis.

Authors do not need to submit their entire data set if only a portion of the data was used in the reported study.

If your submission does not contain these data, please either upload them as Supporting Information files or deposit them to a stable, public repository and provide us with the relevant URLs, DOIs, or accession numbers. For a list of recommended repositories, please see https://journals.plos.org/plosone/s/recommended-repositories.

If there are ethical or legal restrictions on sharing a de-identified data set, please explain them in detail (e.g., data contain potentially sensitive information, data are owned by a third-party organization, etc.) and who has imposed them (e.g., an ethics committee). Please also provide contact information for a data access committee, ethics committee, or other institutional body to which data requests may be sent. If data are owned by a third party, please indicate how others may request data access.

4. Thank you for stating the following in the Acknowledgments Section of your manuscript:

" This work was supported by the United States Department of Agriculture, Animal and Plant Health Inspection Service (USDA APHIS) under grant number AP23VSSPRS00C130. We thank the Texas Parks and Wildlife Department for their productive collaboration on this project. Initial collaborative efforts with Texas Parks and Wildlife were made possible through funding from a Safari Club International Foundationgrant."

We note that you have provided additional information within the Acknowledgements Section that is not currently declared in your Funding Statement. Please note that funding information should not appear in the Acknowledgments section or other areas of your manuscript. We will only publish funding information present in the Funding Statement section of the online submission form.

Please remove any funding-related text from the manuscript and let us know how you would like to update your Funding Statement. Currently, your Funding Statement reads as follows:

" This work was supported by the United States Department of Agriculture, Animal and Plant Health Inspection Service (USDA APHIS) under grant number AP23VSSPRS00C130. LW was also supported through funding from a Safari Club International Foundation grant. The funders had no role in the study design, data collection and analysis, decision to publish, or preparation of the manuscript."

Please include your amended statements within your cover letter; we will change the online submission form on your behalf.

5. Please include captions for your Supporting Information files at the end of your manuscript, and update any in-text citations to match accordingly. Please see our Supporting Information guidelines for more information: http://journals.plos.org/plosone/s/supporting-information.

6. If the reviewer comments include a recommendation to cite specific previously published works, please review and evaluate these publications to determine whether they are relevant and should be cited. There is no requirement to cite these works unless the editor has indicated otherwise.

Additional Editor Comments (if provided):

After careful evaluation by three independent experts in the field, I am pleased to inform you that your manuscript has been recognized as a potentially valuable contribution to chronic wasting disease (CWD) surveillance policy. The reviewers found the study to be well written and the overall approach promising.

However, several substantial concerns have been raised that must be adequately addressed before the manuscript can be considered for publication. Please submit a revised manuscript that addresses all of the points raised, along with a detailed, point-by-point response to the reviewers’ comments.

We look forward to receiving your revised manuscript.

[Note: HTML markup is below. Please do not edit.]

Reviewers' comments:

Reviewer's Responses to Questions

**Comments to the Author**

1. Is the manuscript technically sound, and do the data support the conclusions?

The manuscript must describe a technically sound piece of scientific research with data that supports the conclusions. Experiments must have been conducted rigorously, with appropriate controls, replication, and sample sizes. The conclusions must be drawn appropriately based on the data presented.

Reviewer #1: Partly

Reviewer #2: Partly

Reviewer #3: Partly

**********

2. Has the statistical analysis been performed appropriately and rigorously?

Reviewer #1: N/A

Reviewer #2: N/A

Reviewer #3: Yes

**********

3. Have the authors made all data underlying the findings in their manuscript fully available?

The PLOS Data policy requires authors to make all data underlying the findings described in their manuscript fully available without restriction, with rare exception (please refer to the Data Availability Statement in the manuscript PDF file). The data should be provided as part of the manuscript or its supporting information, or deposited to a public repository. For example, in addition to summary statistics, the data points behind means, medians and variance measures should be available. If there are restrictions on publicly sharing data—e.g. participant privacy or use of data from a third party—those must be specified.

Reviewer #1: No

Reviewer #2: Yes

Reviewer #3: Yes

**********

4. Is the manuscript presented in an intelligible fashion and written in standard English?

PLOS ONE does not copyedit accepted manuscripts, so the language in submitted articles must be clear, correct, and unambiguous. Any typographical or grammatical errors should be corrected at revision, so please note any specific errors here.

Reviewer #1: Yes

Reviewer #2: Yes

Reviewer #3: Yes

**********

5. Review Comments to the Author

Please use the space provided to explain your answers to the questions above. You may also include additional comments for the author, including concerns about dual publication, research ethics, or publication ethics. (Please upload your review as an attachment if it exceeds 20,000 characters)

Reviewer #1: Wakefield et al. “A simulation-based approach to strengthen chronic wasting disease surveillance in captive cervid populations”

The authors have taken historical data from the US state of Texas and performed simulations to determine the likelihood of having detected CWD in negative farms. This tool can be useful for identifying facilities that should increase testing so that they can be confident that negative results in a subset of animals are more likely to be reflective of a CWD negative facility. It remains unclear, however, whether this information could be gleaned from simply looking at the proportion of animals tested in each facility.

Major points:

The requirements of the USDA herd certification program should be clearly stated. It is my understanding that while there is no requirement that a percentage of the heard be tested in a given year, in addition to animals that die at an age greater than 12 months, any animal greater than 12 months old slaughtered on or off site must also be tested. On a farm, one would expect this to be a large number of the heard, or at the very least a relatively consistent percentage from harvest to harvest. This leads into my second point: it would benefit the reader to have a summary of the real-life data associated with each facility in Tables 1 and 2. How many animals were in these facilities in each year, and how many/what proportion were tested? What tests were used? What was the sensitivity of these tests? Without this information, it is difficult to assess the benefit of this modeling. Are these detection probabilities simply a reflection of the proportion of animals tested?

It is unclear from the methodology exactly how animal movement from one facility to another is taken into account. Movement would generally be assumed to entail additional risk to the facility, is this incorporated into the model? Or is this Texas specific, where animals that move must first be tested?

Figure 1: What do the individual data points represent?

The statement on line 85: "Even with the requirement for anti-mortem testing of all cervids prior to movement from a facility, sample sizes are often insufficient to confidently detect CWD in low prevalence scenarios". This requirement, presumably, refers to the additional mandates from the state of Texas. However, if all animals are tested, how could the sample size be insufficient? Please clarify this statement.

Editorial points:

Line 36: “misfolded isoforms” should be changed, as prions are not isoforms of the prion protein. Suggestion of “misfolded forms” or “misfolding of the normal..”

Line 39: CWD has also been detected in Europe and Asia

Line 47: suggest “the US state of South Dakota”

Line 48: suggest “the Canadian province of Saskatchewan”

Line 51: “fully impermeable” suggest “impermeable”

Line 54: “prevalence levels” suggest “prevalence”

Line 57: “infectious prions” suggest “prions”

Line 59: “infection” suggest “infectivity”

Line 61: suggest reference for the environmental persistence of prions

-Johnson, Christopher J., et al. "Prions adhere to soil minerals and remain infectious." PLoS pathogens 2.4 (2006): e32.

Line 289: “infectious prions” suggest “prions”

Reviewer #2: There are two major limitations to this approach. Firstly, cervids do not exhibit positivity in peripheral tissues like the tonsil until 6-18 months post exposure (see Mathiason et al. 2009 and others). This sensitivity issue is compounded when only IHC is used instead of sensitive amplification assays (e.g. real-time quaking induced conversion). This delay to positivity was not accounted for in the simulation, and further, the authors claim a 99% negative predictive value. This is simply impossible with a disease like chronic wasting disease that can take years to manifest. Secondly, the percentage of the herd tested needs to be accounted for in the model. The authors mention the importance of this consideration, yet it was unclear if the percentage of herd tested was accounted for in the model.

Minor comments include the following: authors should consider biological sex as a variable for chronic wasting disease modeling, and figure legend specifies green when the color in the figure appears blue.

Reviewer #3: The manuscript by Wakefield, L. et al (PONE-D-25-67529) entitled, “A simulation-based approach to strengthen chronic wasting disease surveillance in captive cervid populations,” explains the authors’ simulation-based method to analyze CWD surveillance results with the purpose to support monitoring of cervid herds. The study is straightforward and the manuscript is well written. Overall the approach may prove to be useful for CWD surveillance policy. While enthusiasm for the manuscript is high, there are a few concerns the authors should address.

Lines 240-243: As the authors note here, the approach is retrospective in nature. Such a point should be highlighted and discussed throughout the manuscript, beginning in the abstract. The abstract does not clearly state how the approach should be used, but rather summarizes that the approach addresses the issue with negative CWD results. Instead the authors should state early on that this is a retrospective approach that can be applied to historical CWD testing data and be used to identify at-risk herds. Throughout the manuscript, especially the management implementation section, the authors should highlight the application of the approach to identify at risk herds. This is a valuable application of the approach and should be a focus of the authors descript of the use of the application.

Lines 85-87: The authors write, “…sample sizes are often insufficient to confidently detect CWD in low-prevalence scenarios,” but can the authors provide a literature citation to statistical data that support this statement? Have there been any studies that performed power analyses, false discovery rate (q-value), or other statistical analyses to determine the sample size needed for confident determination of CWD presence or absence for a given testing method?

The introduction/background section would benefit from a brief discussion of the CWD testing methods and their sensitivity, as mentioned in the section of Lines 122-141. For example, the RT-QulC test on deer urine other biological samples, like blood or cerebrospinal fluid. Such a discussion would help put the shortcomings of testing in context for the manuscript.

Lines 50-51: Can the authors state this in the positive? For example, “There is a persistent risk of reciprocal CWD transmission between captive and wild cervids, because fences are permeable.”

Lines 142-161: A salient point that the authors should explicitly state is whether testing scenarios re-tested previously tested deer and what proportion of the annual sample test size (e.g. was 1 out of 10 deer previously tested?). Additionally, the authors state that 1,000 iterations were tested. How was each iteration varied between one another? Was a new iteration a random selection, semi-random selection, or non-random selection of tested deer? Finally, how were sampling sizes determined? Is a percentage of the heard tested? It would be helpful if there was some statistical analysis to help determine the testing sample size given the population size (and possibly including the variable of pen size- like 1 sampled deer per 10 deer per acre). If such parameters are already accounted for in CapOvCWD-then please reference these points in this section.

**********

6. PLOS authors have the option to publish the peer review history of their article (what does this mean?). If published, this will include your full peer review and any attached files.

If you choose “no”, your identity will remain anonymous but your review may still be made public.

**Do you want your identity to be public for this peer review?** For information about this choice, including consent withdrawal, please see our Privacy Policy.

Reviewer #1: No

Reviewer #2: No

Reviewer #3: No

**********

[NOTE: If reviewer comments were submitted as an attachment file, they will be attached to this email and accessible via the submission site. Please log into your account, locate the manuscript record, and check for the action link "View Attachments". If this link does not appear, there are no attachment files.]

To ensure your figures meet our technical requirements, please review our figure guidelines: https://journals.plos.org/plosone/s/figures

You may also use PLOS’s free figure tool, NAAS, to help you prepare publication quality figures: https://journals.plos.org/plosone/s/figures#loc-tools-for-figure-preparation.

NAAS will assess whether your figures meet our technical requirements by comparing each figure against our figure specifications.

# Author comment: Author response to Decision Letter 1

**DOI:** 10.1371/journal.pone.0350825.r002

29 Apr 2026

Reviewer #1: Wakefield et al. “A simulation-based approach to strengthen chronic wasting disease surveillance in captive cervid populations”

The authors have taken historical data from the US state of Texas and performed simulations to determine the likelihood of having detected CWD in negative farms. This tool can be useful for identifying facilities that should increase testing so that they can be confident that negative results in a subset of animals are more likely to be reflective of a CWD negative facility. It remains unclear, however, whether this information could be gleaned from simply looking at the proportion of animals tested in each facility.

Response: Thank you for the thorough review. We have addressed your concerns and incorporated most of the suggestions.

Major points:

The requirements of the USDA herd certification program should be clearly stated. It is my understanding that while there is no requirement that a percentage of the heard be tested in a given year, in addition to animals that die at an age greater than 12 months, any animal greater than 12 months old slaughtered on or off site must also be tested.

Response: USDA herd certification program requirements are discussed here:

Lines 78: A key requirement of this program is that all on-farm deaths of cervids aged 12 months or older must be tested for CWD. Five consecutive years of CWD-free testing is required to certify herds as being low risk for having CWD, and animals from such herds may be shipped interstate.

Also, line 86: Individual states may implement regulations that are more stringent than the HCP requirements. For example, in Texas, in addition to requiring CWD testing of all mortalities within captive cervid facilities, ante-mortem testing is also mandated for any deer being moved from a facility.

On a farm, one would expect this to be a large number of the heard, or at the very least a relatively consistent percentage from harvest to harvest.

Response: The number of animals tested in captive cervid facilities is generally low (see new table 1). Under the USDA Herd Certification Program, only on-farm mortalities of cervids older than 12 months are required to be tested for CWD, and in Texas, ante-mortem testing is additionally required for deer moved out of a facility. Consequently, only a subset of the herd is tested in any given year. In facilities with low mortality, small herd size, or limited animal movement, the number of deer tested may be very small and, in some years, no deer may be tested at all.

This leads into my second point: it would benefit the reader to have a summary of the real-life data associated with each facility in Tables 1 and 2. How many animals were in these facilities in each year, and how many/what proportion were tested?

Response: New table 1 added: proportion of deer tested

What tests were used? What was the sensitivity of these tests? Without this information, it is difficult to assess the benefit of this modeling. Are these detection probabilities simply a reflection of the proportion of animals tested?

Response: Line 147: In addition to the required post-mortem testing of retropharyngeal lymph nodes and brainstem tissue using IHC or ELISA, TPWD and the Texas Animal Health Commission (TAHC) have incorporated ante-mortem testing using immunohistochemistry (IHC) of samples obtained via tonsil biopsy or rectal biopsy.

The number of deer tested in the captive facilities may be very small and, in some years, no deer may be tested at all (See new Table 1). We estimate the facility-level probability of detecting a single infected deer given the sampling intensity achieved and the diagnostic tests currently in use.

It is unclear from the methodology exactly how animal movement from one facility to another is taken into account. Movement would generally be assumed to entail additional risk to the facility, is this incorporated into the model? Or is this Texas specific, where animals that move must first be tested?

Response: Animal movement between facilities is incorporated through the individual deer transfer histories used to initialize the model for each accounting year. For each facility, the Texas Parks and Wildlife Department maintains annual records of herd size, herd composition, transfers into and out of the facility, and individual deer testing histories. These data are used to construct the model population for each accounting year.

Any deer that was present in a facility at any point during a given accounting year is included in that year’s model population, even if the animal was later transferred or died before the end of the year. Thus, deer moved into a facility contribute to the population at risk during the year they were present, and deer moved out remain part of the facility’s annual risk profile for that year.

We intentionally adopted this approach to represent a conservative, “worst-case” scenario for estimating the likelihood of undetected CWD. Because an infected deer may have been present in the facility for only part of the year before being transferred or dying, excluding such individuals could underestimate the facility-level risk of undetected CWD.

See lines145-149: Deer that were present in the facility at any point during an accounting year are included in that year’s model population, including individuals that died or were transferred in or out before the end of the year. These deer are included in the model to represent a “worst case scenario” for facilities to estimate the risk of undetected CWD in the facility.

Figure 1: What do the individual data points represent?

Response: The individual data points represent annual CWD detection probabilities (y-axis) for a given accounting year (x-axis). Annual detection probability is estimated as the proportion of model iterations in which CWD-infected deer is included in the tested sample, thereby resulting in successful detection of CWD within the facility. For each accounting year, 1000 iterations were conducted and summarized into 10 replicate detection probability estimates (each based on 100 iterations), which are shown as the individual data points.

Line 172 changed: Annual CWD detection probability was estimated as the proportion of iterations in which the CWD-infected deer was included in the tested sample, thereby resulting in successful detection of CWD.

Line 191: For each accounting year, 1000 iterations were conducted and summarized into 10 replicate detection probability estimates (each based on 100 iterations).

The statement on line 85: "Even with the requirement for anti-mortem testing of all cervids prior to movement from a facility, sample sizes are often insufficient to confidently detect CWD in low prevalence scenarios". This requirement, presumably, refers to the additional mandates from the state of Texas. However, if all animals are tested, how could the sample size be insufficient? Please clarify this statement.

Response: All animals in a facility are not tested - under the USDA Herd Certification Program, only on-farm mortalities of cervids older than 12 months are required to be tested for CWD, and in Texas, ante-mortem testing is additionally required for deer moved out of a facility. Consequently, only a subset of the herd is tested in any given year. In facilities with low mortality, small herd size, or limited animal movement, the number of deer tested may be very small and, in some years, no deer may be tested at all. Even when all required animals test “CWD not detected,” these animals may represent only a small proportion of the herd. The central question addressed in this paper is what such negative results imply about the likelihood that undetected CWD may still be present in the remainder of the facility.

Editorial points:

Line 36: “misfolded isoforms” should be changed, as prions are not isoforms of the prion protein. Suggestion of “misfolded forms” or “misfolding of the normal..”

Response: Changed to ‘..misfolded forms of the normal host cellular prion protein.’

Line 39: CWD has also been detected in Europe and Asia

Response: Added the following: CWD has also been detected in free-ranging reindeer (Rangifer tarandus) in Norway and in captive cervids in South Korea. Moreover, a novel form of CWD with atypical characteristics has been reported in moose and red deer in Norway, as well as in moose in Finland and Sweden (Pirisinu et al. 2018, Vikøren et al. 2019, Mysterud et al. 2020, Hopp et al. 2024). These cases appear to represent sporadic CWD (sCWD), with little or no evidence of transmission between live animals.

Hopp P, Rolandsen CM, Korpenfelt S-L, Våge J, Sörén K, Solberg EJ, Averhed G, Pusenius J, Rosendal T, Ericsson G, Bakka HC, Mysterud A, Gavier-Widén D, Hautaniemi M, Ågren E, Isomursu M, Madslien K, Benestad SL, Nöremark M. 2024. Sporadic cases of chronic wasting disease in old moose – an epidemiological study. Journal of General Virology 105.

Mysterud A, Benestad SL, Rolandsen CM, Våge J. 2020. Policy implications of an expanded chronic wasting disease universe. Journal of Applied Ecology 58: 281–285.

Pirisinu L, Tran L, Chiappini B, Vanni I, Di Bari MA, Vaccari G, Vikøren T, Madslien KI, Våge J, Spraker T, Mitchell G, Balachandran A, Baron T, Casalone C, Rolandsen CM, Røed KH, Agrimi U, Nonno R, Benestad SL. 2018. Novel Type of Chronic Wasting Disease Detected in Moose (Alces alces), Norway. Emerging Infectious Diseases 24: 2210–2218.

Vikøren T, Våge J, Madslien KI, Røed KH, Rolandsen CM, Tran L, Hopp P, Veiberg V, Heum M, Moldal T, Neves CGD, Handeland K, Ytrehus B, Kolbjørnsen Ø, Wisløff H, Terland R, Saure B, Dessen KM, Svendsen SG, Nordvik BS, Benestad SL. 2019. First Detection of Chronic Wasting Disease in a Wild Red Deer (Cervus elaphus) in Europe. Journal of Wildlife Diseases 55: 970.

Line 47: suggest “the US state of South Dakota”

Response: Changed to ‘..from the US state of South Dakota..’

Line 48: suggest “the Canadian province of Saskatchewan”

Response: Changed to ‘…the Canadian province of Saskatchewan…’

Line 51: “fully impermeable” suggest “impermeable”

Response: Changed to ‘Moreover, there is a persistent risk of reciprocal CWD transmission between captive and wild cervids, because fences are permeable (Gerhold & Hickling, 2016).’

Line 54: “prevalence levels” suggest “prevalence”

Response: Changed to ‘..and in some cases, prevalence nearing 100%’

Line 57: “infectious prions” suggest “prions”

Response: Contamination is due to the prions shed by the CWD+ deer that are, and remain infectious in the environment, hence infectious prions.

Line 59: “infection” suggest “infectivity”

Response: Changed to ‘..creating reservoirs of infectivity..’

Line 61: suggest reference for the environmental persistence of prions

-Johnson, Christopher J., et al. "Prions adhere to soil minerals and remain infectious." PLoS pathogens 2.4 (2006): e32.

Response: Reference added: Johnson CJ, Phillips KE, Schramm PT, McKenzie D, Aiken JM, Pedersen JA. 2006. Prions adhere to soil minerals and remain infectious. PLoS Pathogens 2: e32.

Line 289: “infectious prions” suggest “prions”

Response: The prions shed by the CWD+ deer are, and remain infectious in the environment, hence infectious prions.

Reviewer #2: There are two major limitations to this approach. Firstly, cervids do not exhibit positivity in peripheral tissues like the tonsil until 6-18 months post exposure (see Mathiason et al. 2009 and others). This sensitivity issue is compounded when only IHC is used instead of sensitive amplification assays (e.g. real-time quaking induced conversion). This delay to positivity was not accounted for in the simulation, and further, the authors claim a 99% negative predictive value. This is simply impossible with a disease like chronic wasting disease that can take years to manifest.

Response: The reviewer’s point regarding delay to positivity is logical, but it does not directly apply to the purpose of this analysis. We are not using this approach to certify that a facility is free of CWD. Rather, we estimate the facility-level probability of detecting a single infected deer given the sampling intensity achieved and the diagnostic tests currently in use. Thus, the approach is designed to evaluate the confidence that can be placed in existing surveillance rather than to declare disease freedom.

Our approach provides a practical means of interpreting “CWD not detected” results from a subset of the herd by accounting for herd size, individual deer domiciliary history, and CWD testing records. In doing so, it offers regulatory agencies a decision-support tool for standardizing surveillance across captive cervid facilities and promoting more sustainable and efficient CWD surveillance strategies.

Also, the comment that a 99% negative predictive value (NPV) is impossible is not correct. Unlike diagnostic sensitivity, which is an intrinsic property of a test, NPV depends strongly on the pre-test probability of disease and therefore increases as disease prevalence decreases. In low-prevalence situations such as those considered here (<1%), even diagnostic tests with relatively modest sensitivity (e.g., 25–70%) can have NPVs exceeding 99%, because the large majority of animals tested are truly uninfected. This relationship is well established in diagnostic epidemiology; see, for example, Monaghan et al. 2021. 10.3390/medicina57050503

Secondly, the percentage of the herd tested needs to be accounted for in the model. The authors mention the importance of this consideration, yet it was unclear if the percentage of herd tested was accounted for in the model.

Response: Yes, the proportion of the herd tested is indeed accounted for in this agent-based model. Please refer to line 133: CapOvCWD was initialized using data on herd size, composition, and individual deer transfer histories obtained from captive facility records maintained by the Texas Parks and Wildlife Department (TPWD).

Line 145: The number of adult and fawn deer tested for CWD in each accounting year was determined from individual testing histories, which included both ante-mortem and post-mortem test results recorded within the accounting year.

Minor comments include the following: authors should consider biological sex as a variable for chronic wasting disease modeling, and figure legend specifies green when the color in the figure appears blue.

Response: This approach does not really model chronic wasting disease, we are just simulating CWD surveillance scenarios in captive facilities to determine facility-level CWD detection probabilities. Biological sex can be readily incorporated in the model for estimating CWD detection probabilities.

Changed figure caption: Teal colored boxes indicate detection probabilities based on annual testing data, while red colored boxes represent refined estimates derived from multiyear testing histories (“Lifetime testing”) of individual deer.

Reviewer #3: The manuscript by Wakefield, L. et al (PONE-D-25-67529) entitled, “A simulation-based approach to strengthen chronic wasting disease surveillance in captive cervid populations,” explains the authors’ simulation-based method to analyze CWD surveillance results with the purpose to support monitoring of cervid herds. The study is straightforward and the manuscript is well written. Overall the approach may prove to be useful for CWD surveillance policy. While enthusiasm for the manuscript is high, there are a few concerns the authors should address.

Lines 240-243: As the authors note here, the approach is retrospective in nature. Such a point should be highlighted and discussed throughout the manuscript, beginning in the abstract. The abstract does not clearly state how the approach should be used, but rather summarizes that the approach addresses the issue with negative CWD results. Instead the authors should state early on that this is a retrospective approach that can be applied to historical CWD testing data and be used to identify at-risk herds. Throughout the manuscript, especially the management implementation section, the authors sho

Attachment Submitted filename: Response_to_reviewers.docx

# Peer review: Decision Letter 1

**DOI:** 10.1371/journal.pone.0350825.r003

**Authors:** Jung-Yong Yeh

17 May 2026

PONE-D-25-67529R1

A simulation-based approach to strengthen chronic wasting disease surveillance in captive cervid populations

PLOS One

Dear Dr. Belsare,

Thank you for submitting your manuscript to PLOS ONE. After careful consideration, we feel that it has merit but does not fully meet PLOS ONE’s publication criteria as it currently stands. Therefore, we invite you to submit a revised version of the manuscript that addresses the points raised during the review process.

Please submit your revised manuscript by Jul 01 2026 11:59PM. If you will need more time than this to complete your revisions, please reply to this message or contact the journal office at plosone@plos.org. When you're ready to submit your revision, log on to https://www.editorialmanager.com/pone/ and select the 'Submissions Needing Revision' folder to locate your manuscript file.

Please include the following items when submitting your revised manuscript:

- A letter that responds to each point raised by the academic editor and reviewer(s). You should upload this letter as a separate file labeled 'Response to Reviewers'.
- A marked-up copy of your manuscript that highlights changes made to the original version. You should upload this as a separate file labeled 'Revised Manuscript with Track Changes'.
- An unmarked version of your revised paper without tracked changes. You should upload this as a separate file labeled 'Manuscript'.

If you would like to make changes to your financial disclosure, please include your updated statement in your cover letter. Guidelines for resubmitting your figure files are available below the reviewer comments at the end of this letter.

If applicable, we recommend that you deposit your laboratory protocols in protocols.io to enhance the reproducibility of your results. Protocols.io assigns your protocol its own identifier (DOI) so that it can be cited independently in the future. For instructions see: https://journals.plos.org/plosone/s/submission-guidelines#loc-laboratory-protocols. Additionally, PLOS ONE offers an option for publishing peer-reviewed Lab Protocol articles, which describe protocols hosted on protocols.io. Read more information on sharing protocols at https://plos.org/protocols?utm_medium=editorial-email&utm_source=authorletters&utm_campaign=protocols.

As the corresponding author, your ORCID iD is verified in the submission system and will appear in the published article. PLOS supports the use of ORCID, and we encourage all coauthors to register for an ORCID iD and use it as well. Please encourage your coauthors to verify their ORCID iD within the submission system before final acceptance, as unverified ORCID iDs will not appear in the published article. *Only* the individual author can complete the verification step; PLOS staff *cannot* verify ORCID iDs on behalf of authors.

We look forward to receiving your revised manuscript.

Kind regards,

Jung-Yong Yeh

Academic Editor

PLOS One

**Journal Requirements:**

If the reviewer comments include a recommendation to cite specific previously published works, please review and evaluate these publications to determine whether they are relevant and should be cited. There is no requirement to cite these works unless the editor has indicated otherwise.

**Additional Editor Comments:**

The reviewers identified several areas requiring clarification, further development, and correction. I therefore invite the authors to revise the manuscript in accordance with the reviewers’ comments and to submit a revised version together with a detailed point-by-point response addressing each issue raised.

[Note: HTML markup is below. Please do not edit.]

Reviewers' comments:

Reviewer's Responses to Questions

**Comments to the Author**

1. If the authors have adequately addressed your comments raised in a previous round of review and you feel that this manuscript is now acceptable for publication, you may indicate that here to bypass the “Comments to the Author” section, enter your conflict of interest statement in the “Confidential to Editor” section, and submit your "Accept" recommendation.

Reviewer #1: All comments have been addressed

Reviewer #2: (No Response)

Reviewer #3: All comments have been addressed

**********

2. Is the manuscript technically sound, and do the data support the conclusions?

The manuscript must describe a technically sound piece of scientific research with data that supports the conclusions. Experiments must have been conducted rigorously, with appropriate controls, replication, and sample sizes. The conclusions must be drawn appropriately based on the data presented.

Reviewer #1: Yes

Reviewer #2: Partly

Reviewer #3: Yes

**********

3. Has the statistical analysis been performed appropriately and rigorously?

Reviewer #1: Yes

Reviewer #2: N/A

Reviewer #3: Yes

**********

4. Have the authors made all data underlying the findings in their manuscript fully available?

The PLOS Data policy requires authors to make all data underlying the findings described in their manuscript fully available without restriction, with rare exception (please refer to the Data Availability Statement in the manuscript PDF file). The data should be provided as part of the manuscript or its supporting information, or deposited to a public repository. For example, in addition to summary statistics, the data points behind means, medians and variance measures should be available. If there are restrictions on publicly sharing data—e.g. participant privacy or use of data from a third party—those must be specified.

Reviewer #1: Yes

Reviewer #2: Yes

Reviewer #3: Yes

**********

5. Is the manuscript presented in an intelligible fashion and written in standard English?

PLOS ONE does not copyedit accepted manuscripts, so the language in submitted articles must be clear, correct, and unambiguous. Any typographical or grammatical errors should be corrected at revision, so please note any specific errors here.

Reviewer #1: Yes

Reviewer #2: Yes

Reviewer #3: Yes

**********

6. Review Comments to the Author

Please use the space provided to explain your answers to the questions above. You may also include additional comments for the author, including concerns about dual publication, research ethics, or publication ethics. (Please upload your review as an attachment if it exceeds 20,000 characters)

**Reviewer #1:** The authors have responded adequately to my initial critiques. My suggestion that “infectious prions” be changed to “prions” is rooted in the fact that prions are, by definition, infectious, making the statement “infectious prions” redundant. I will not belabour this point further, however.

The inclusion of the new table 1, which provides the proportion of animals tested in each year, reiterates a point made in the first round of review. At first glance, it appears that the model is simply assigning confidence to years in which a very large proportion of animals were tested. For example, in 2023 TxCF1-4 had the following proportions of animals tested: 208/260, 237/265, 423/477, 41/6, 227/288. Looking at these numbers alone, I would conclude that it is unlikely that CWD was present in these facilities, and it is unclear to me what additional insight is provided by this modeling in these scenarios. In the case of TxCF18-19, there is high confidence that fawns in 2020 did not have CWD despite no fawns being tested. In other instances in 2023, the confidence is low using multi-year data despite relatively high testing percentages (TxCF5, 11, 13). I feel the manuscript would be improved if this, and potentially other examples, are explicitly discussed in this light to further highlight the usefulness of this modeling.

**Reviewer #2:** The authors did not address limitations pointed out previously. Concise recommendations are provided below.

As the authors point out, a 99% negative predictive value can be achieved from low prevalence scenarios. However, the current verbiage is "Across this sensitivity range, the NPV exceeded 99%, indicating a high likelihood that deer with CWD not detected results using ante-mortem methods were free of CWD, supporting the inclusion of CWD not detected ante-mortem tests as reliable indicators of CWD-negative status in the model.” Change to “Across this sensitivity range and prevalence <1%, the NPV exceeded 99%, indicating a high likelihood that deer with CWD not detected results using ante-mortem methods were free of CWD, supporting the inclusion of CWD not detected ante-mortem tests as reliable indicators of CWD-negative status in the model. However, NPV will decrease as prevalence increases, and as such, the 99% NPV value is only applicable for low prevalence scenarios” or something similar. This should be abundantly clear in the manuscript.

At a minimum, authors should mention the unique aspects of CWD pathogenesis that lead to delays in positivity, even for antemortem samples. Their simulation relies on gold standard diagnostic tests that require months post exposure to show positivity. This must be mentioned in the manuscript as a limitation to data interpretation. Specifically, a negative test result could be interpreted as the animal is truly CWD-free or the animal has been exposed but has not reached the threshold to positivity. For example, "... a CWD not detected test result indicates that the deer was not infected in any previous year" is simply not accurate.

In the response to reviewer comments, the authors mention “…the approach is designed to evaluate the confidence that can be placed in existing surveillance rather than to declare disease freedom.” As written, the manuscript does not reflect this. For example, "... a high likelihood that deer with CWD not detected results using ante-mortem methods were free of CWD, supporting the inclusion of CWD not detected ante-mortem test results as reliable indicators of CWD-negative status in the model." Further, "..CapOvCWD described here provides a mechanism for assessing the comprehensive risk of undetected CWD in captive cervid facilities."

Lastly, as presented, it is still unclear whether the percentage of herd tested was explicitly included in the simulation. The authors refer to two separate lines that point out these variables, but it would be helpful to make this more concise.

**Reviewer #3:** (No Response)

**********

7. PLOS authors have the option to publish the peer review history of their article (what does this mean?). If published, this will include your full peer review and any attached files.

If you choose “no”, your identity will remain anonymous but your review may still be made public.

**Do you want your identity to be public for this peer review?** For information about this choice, including consent withdrawal, please see our Privacy Policy.

Reviewer #1: No

Reviewer #2: No

Reviewer #3: No

**********

[NOTE: If reviewer comments were submitted as an attachment file, they will be attached to this email and accessible via the submission site. Please log into your account, locate the manuscript record, and check for the action link "View Attachments". If this link does not appear, there are no attachment files.]

To ensure your figures meet our technical requirements, please review our figure guidelines: https://journals.plos.org/plosone/s/figures

You may also use PLOS’s free figure tool, NAAS, to help you prepare publication quality figures: https://journals.plos.org/plosone/s/figures#loc-tools-for-figure-preparation.

NAAS will assess whether your figures meet our technical requirements by comparing each figure against our figure specifications.

# Author comment: Author response to Decision Letter 2

**DOI:** 10.1371/journal.pone.0350825.r004

18 May 2026

05/18/2026

Response to reviewers

We are grateful to the reviewers and the editor for their comments and suggestions. Along with the revised manuscript, we provide this detailed response to reviewer comments. Our responses to reviewer comments are in blue. Changes in response to the comments in the revised manuscript are in red.

Reviewer #1: The authors have responded adequately to my initial critiques. My suggestion that “infectious prions” be changed to “prions” is rooted in the fact that prions are, by definition, infectious, making the statement “infectious prions” redundant. I will not belabour this point further, however.

Response: Line 63: Infectious prions changed to prions.

---

The inclusion of the new table 1, which provides the proportion of animals tested in each year, reiterates a point made in the first round of review. At first glance, it appears that the model is simply assigning confidence to years in which a very large proportion of animals were tested. For example, in 2023 TxCF1-4 had the following proportions of animals tested: 208/260, 237/265, 423/477, 41/61, 227/288. Looking at these numbers alone, I would conclude that it is unlikely that CWD was present in these facilities, and it is unclear to me what additional insight is provided by this modeling in these scenarios.

Response: The annual CWD detection probabilities estimated using the proportion of animals tested every year (Table 1) are presented in Table 2. Additional insights provided by the modeling approach are in Table 3. For the 5 facilities highlighted by Reviewer 1 (TxCF1-5), note that increased testing resulted in increased CWD detection probability (> 80%) only in the year when more deer were tested (2023 for TxCF1,2,3,5 and 2021 for TxCF4) - see Table 2. But for the same data, our modeling approach provides greater improvements in detection probability estimates across years – see table 3.

Please check line270: Facility TxCF2 illustrates this relationship particularly well. Between 2014 and 2024, annual CWD testing of adult deer (both antemortem and postmortem) ranged from 2% to 26% of the herd, with the exception of 2021 and 2023, when testing reached 40% and 80%, respectively. As expected, the annual CWD detection probabilities based solely on testing conducted within a given accounting year remained below 25%, except in the two years with larger sample sizes. In contrast, the refined detection probability estimates—calculated using the cumulative number of confirmed CWD-negative individuals present in the facility during an accounting year—were substantially higher (>40%). Notably, during the three-year period from 2021 to 2023, the refined estimates exceeded 90%, reflecting the impact of intensified sampling and cumulative testing history. This indicates a low likelihood (<10%) of undetected CWD in the facility during those years. Facility TxCF2, along with others that achieved similarly high refined annual CWD detection probabilities (e.g., TxCF3, TxCF4, TxCF5, and TxCF17), may serve as benchmarks for meaningful and sustainable CWD surveillance in captive cervid populations.

---

In the case of TxCF18-19, there is high confidence that fawns in 2020 did not have CWD despite no fawns being tested.

Response: Exactly, the modeling approach provides high confidence despite low/no testing - this is explained in the manuscript:

Line 255: Pooling CWD testing data over multiple years for individual deer enables us to refine and optimize CWD detection probability estimates retrospectively. This longitudinal approach accounts for the CWD status of deer that may have been present in a facility during a given year but were not tested for CWD until a later year. CWD testing can occur either in the same facility or another, and may be conducted either antemortem or postmortem, allowing for a more comprehensive understanding of the risk of undetected CWD over time.

---

In other instances in 2023, the confidence is low using multi-year data despite relatively high testing percentages (TxCF5, 11, 13). I feel the manuscript would be improved if this, and potentially other examples, are explicitly discussed in this light to further highlight the usefulness of this modeling.

Response: Please also check line 297: For instance, facilities TxCF13, TxCF14, TxCF16, and TxCF19 all had annual CWD detection probabilities for adults below 70%, particularly in the years following 2020. Prioritizing these facilities for increased CWD surveillance efforts could reduce the likelihood of undetected CWD and provide greater confidence that CWD is truly absent from these facilities.

----

Reviewer #2:

As the authors point out, a 99% negative predictive value can be achieved from low prevalence scenarios. However, the current verbiage is "Across this sensitivity range, the NPV exceeded 99%, indicating a high likelihood that deer with CWD not detected results using ante-mortem methods were free of CWD, supporting the inclusion of CWD not detected ante-mortem tests as reliable indicators of CWD-negative status in the model.” Change to “Across this sensitivity range and prevalence <1%, the NPV exceeded 99%, indicating a high likelihood that deer with CWD not detected results using ante-mortem methods were free of CWD, supporting the inclusion of CWD not detected ante-mortem tests as reliable indicators of CWD-negative status in the model. However, NPV will decrease as prevalence increases, and as such, the 99% NPV value is only applicable for low prevalence scenarios” or something similar. This should be abundantly clear in the manuscript.

Response: Line 152 changed as follows:

Across this sensitivity range under a low prevalence scenario, NPV exceeded 99%, indicating a high likelihood that deer with CWD not detected ante-mortem test results were free of CWD within the limitations of currently available diagnostics. This supports the use of ante-mortem CWD not detected results as reliable indicators of CWD-negative status within the model framework. Although NPV declines at higher prevalence levels when test sensitivity is low, falling below 90% when prevalence exceeds 10%, surveillance at such prevalence levels requires substantially smaller sample sizes for confident detection of CWD, reducing the practical need for this modeling approach.

---

At a minimum, authors should mention the unique aspects of CWD pathogenesis that lead to delays in positivity, even for antemortem samples. Their simulation relies on gold standard diagnostic tests that require months post exposure to show positivity. This must be mentioned in the manuscript as a limitation to data interpretation. Specifically, a negative test result could be interpreted as the animal is truly CWD-free or the animal has been exposed but has not reached the threshold to positivity.

Response: Added the following: Line 101: At present, no diagnostic test can reliably determine that an individual animal is free of CWD. Existing tests are not sufficiently sensitive to detect all infected animals, particularly during the early stages of infection, because CWD has a prolonged incubation period that can result in false-negative test outcomes before prion accumulation reaches detectable levels.

---

For example, "... a CWD not detected test result indicates that the deer was not infected in any previous year" is simply not accurate.

Response: Changed as follows: Line 171: Therefore, within the limitations of currently available diagnostics, a CWD not detected test result indicates a high likelihood that the deer was not infected in previous years.

---

In the response to reviewer comments, the authors mention “…the approach is designed to evaluate the confidence that can be placed in existing surveillance rather than to declare disease freedom.” As written, the manuscript does not reflect this. For example, "... a high likelihood that deer with CWD not detected results using ante-mortem methods were free of CWD, supporting the inclusion of CWD not detected ante-mortem test results as reliable indicators of CWD-negative status in the model." Further, "..CapOvCWD described here provides a mechanism for assessing the comprehensive risk of undetected CWD in captive cervid facilities."

Response: Changed as above:

Line 152: Across this sensitivity range under a low prevalence scenario, NPV exceeded 99%, indicating a high likelihood that deer with CWD not detected ante-mortem test results were free of CWD within the limitations of currently available diagnostics. This supports the use of ante-mortem CWD not detected results as reliable indicators of CWD-negative status within the model framework.

Please consider this as well:

Line 332: CapOvCWD can serve as a decision-support tool to standardize surveillance across captive cervid facilities, prioritize facilities for enhanced surveillance, and support more sustainable, efficient, and risk-based CWD monitoring. It can also complement existing surveillance programs by identifying gaps and helping agencies target limited surveillance resources more effectively.

---

Lastly, as presented, it is still unclear whether the percentage of herd tested was explicitly included in the simulation. The authors refer to two separate lines that point out these variables, but it would be helpful to make this more concise.

Response: CapOvCWD (the model described in this paper) is an agent-based simulation model. The model initializes a captive deer population (the denominator) and simulates testing of a subset of this population (numerator).

To clarify, the herd (denominator) is explained in line 134: Deer that were present in the facility at any point during an accounting year are included in that year’s model population, including individuals that died or were transferred in or out before the end of the year.

The numerator (sample size or the subset of this population tested for CWD) is explained in line 141: The number of adult and fawn deer tested for CWD in each accounting year was determined from individual testing histories, which included both ante-mortem and post-mortem test results recorded within the accounting year.

Line 165: sample size was defined as the number of deer in the captive facility that were tested during an accounting year and had “CWD not detected” results,

Line 167: During each model iteration, one deer from the relevant age class was randomly designated as CWD-infected, and a sample of the specified size was then randomly selected for CWD testing.

Attachment Submitted filename: Response to Reviewers.docx

# Peer review: Decision Letter 2

**DOI:** 10.1371/journal.pone.0350825.r005

**Authors:** Jung-Yong Yeh

20 May 2026

A simulation-based approach to strengthen chronic wasting disease surveillance in captive cervid populations

PONE-D-25-67529R2

Dear Dr. Belsare,

We’re pleased to inform you that your manuscript has been judged scientifically suitable for publication and will be formally accepted for publication once it meets all outstanding technical requirements.

Within one week, you’ll receive an e-mail detailing the required amendments. When these have been addressed, you’ll receive a formal acceptance letter and your manuscript will be scheduled for publication.

An invoice will be generated when your article is formally accepted. Please note, if your institution has a publishing partnership with PLOS and your article meets the relevant criteria, all or part of your publication costs will be covered. Please make sure your user information is up-to-date by logging into Editorial Manager at Editorial Manager® and clicking the ‘Update My Information' link at the top of the page. For questions related to billing, please contact billing support.

If your institution or institutions have a press office, please notify them about your upcoming paper to help maximize its impact. If they’ll be preparing press materials, please inform our press team as soon as possible -- no later than 48 hours after receiving the formal acceptance. Your manuscript will remain under strict press embargo until 2 pm Eastern Time on the date of publication. For more information, please contact onepress@plos.org.

Kind regards,

Jung-Yong Yeh

Academic Editor

PLOS One

Additional Editor Comments (optional):

The authors have addressed all previous comments and improved the manuscript accordingly. I recommend acceptance for publication.

Reviewers' comments:

Reviewer's Responses to Questions

**Comments to the Author**

1. If the authors have adequately addressed your comments raised in a previous round of review and you feel that this manuscript is now acceptable for publication, you may indicate that here to bypass the “Comments to the Author” section, enter your conflict of interest statement in the “Confidential to Editor” section, and submit your "Accept" recommendation.

Reviewer #2: All comments have been addressed

**********

2. Is the manuscript technically sound, and do the data support the conclusions?

The manuscript must describe a technically sound piece of scientific research with data that supports the conclusions. Experiments must have been conducted rigorously, with appropriate controls, replication, and sample sizes. The conclusions must be drawn appropriately based on the data presented.

Reviewer #2: Yes

**********

3. Has the statistical analysis been performed appropriately and rigorously?

Reviewer #2: N/A

**********

4. Have the authors made all data underlying the findings in their manuscript fully available?

The PLOS Data policy requires authors to make all data underlying the findings described in their manuscript fully available without restriction, with rare exception (please refer to the Data Availability Statement in the manuscript PDF file). The data should be provided as part of the manuscript or its supporting information, or deposited to a public repository. For example, in addition to summary statistics, the data points behind means, medians and variance measures should be available. If there are restrictions on publicly sharing data—e.g. participant privacy or use of data from a third party—those must be specified.

Reviewer #2: Yes

**********

5. Is the manuscript presented in an intelligible fashion and written in standard English?

PLOS ONE does not copyedit accepted manuscripts, so the language in submitted articles must be clear, correct, and unambiguous. Any typographical or grammatical errors should be corrected at revision, so please note any specific errors here.

Reviewer #2: Yes

**********

6. Review Comments to the Author

Please use the space provided to explain your answers to the questions above. You may also include additional comments for the author, including concerns about dual publication, research ethics, or publication ethics. (Please upload your review as an attachment if it exceeds 20,000 characters)

Reviewer #2: All concerns were addressed in the most recent round of edits. Hopefully the CWD antemortem tests improve in the future!

**********

7. PLOS authors have the option to publish the peer review history of their article (what does this mean?). If published, this will include your full peer review and any attached files.

If you choose “no”, your identity will remain anonymous but your review may still be made public.

**Do you want your identity to be public for this peer review?** For information about this choice, including consent withdrawal, please see our Privacy Policy.

Reviewer #2: No

**********

# Editor report: Acceptance letter

**DOI:** 10.1371/journal.pone.0350825.r006

**Authors:** Jung-Yong Yeh

PONE-D-25-67529R2

PLOS One

Dear Dr. Belsare,

I'm pleased to inform you that your manuscript has been deemed suitable for publication in PLOS One. Congratulations! Your manuscript is now being handed over to our production team.

At this stage, our production department will prepare your paper for publication. This includes ensuring the following:

* All references, tables, and figures are properly cited

* All relevant supporting information is included in the manuscript submission,

* There are no issues that prevent the paper from being properly typeset

You will receive further instructions from the production team, including instructions on how to review your proof when it is ready. Please keep in mind that we are working through a large volume of accepted articles, so please give us a few days to review your paper and let you know the next and final steps.

Lastly, if your institution or institutions have a press office, please let them know about your upcoming paper now to help maximize its impact. If they'll be preparing press materials, please inform our press team within the next 48 hours. Your manuscript will remain under strict press embargo until 2 pm Eastern Time on the date of publication. For more information, please contact onepress@plos.org.

You will receive an invoice from PLOS for your publication fee after your manuscript has reached the completed accept phase. If you receive an email requesting payment before acceptance or for any other service, this may be a phishing scheme. Learn how to identify phishing emails and protect your accounts at https://explore.plos.org/phishing.

If we can help with anything else, please email us at customercare@plos.org.

Thank you for submitting your work to PLOS ONE and supporting open access.

Kind regards,

PLOS ONE Editorial Office Staff

on behalf of

Dr. Jung-Yong Yeh

Academic Editor

PLOS One
